# A novel compound heterozygous missense mutation in *ASNS* broadens the spectrum of asparagine synthetase deficiency

**DOI:** 10.1002/mgg3.1235

**Published:** 2020-04-07

**Authors:** Chun Wang, Guiyuan He, Yusong Ge, Runjie Li, Zhenguo Li, Yongzhong Lin

**Affiliations:** ^1^ Department of Neurology The Second Hospital of Dalian Medical University Dalian China; ^2^ Center for Reproductive and Genetic Medicine Dalian Municipal Women and Children’s Medical Center Dalian China; ^3^ Department of Rehabilitation Dalian Municipal Women and Children’s Medical Center Dalian China; ^4^ Clinical Laboratory The Second Hospital of Dalian Medical University Dalian China

**Keywords:** *ASNS*, asparagine synthetase deficiency, bioinformatics analyses, novel mutation, whole exome sequencing

## Abstract

**Background:**

Asparagine synthetase deficiency (ASNSD) is a rare pediatric congenital disorder that clinically manifests into severe progressive microcephaly, global developmental delay, spastic quadriplegia, and refractory seizures. ASNSD is caused by inheritable autosomal recessive mutations in the asparagine synthetase (*ASNS*) gene.

**Methods:**

We performed whole‐exome sequencing using the patient's peripheral blood, and newly discovered mutations were subsequently verified in the patient's parents via Sanger sequencing. Software‐based bioinformatics analyses (protein sequence conservation analysis, prediction of protein phosphorylation sites, protein structure modeling, and protein stability prediction) were performed to investigate and deduce their downstream effects.

**Results:**

In this article, we summarized all the previously reported cases of ASNSD and that of a Chinese girl who was clinically diagnosed with ASNSD, which was later confirmed via genetic testing. Whole‐exome sequencing revealed two compound heterozygous missense mutations within the *ASNS* (c.368T > C, p.F123S and c.1649G > A, p.R550H). The origin of the two mutations was also verified in the patient's parents via Sanger sequencing. The mutation c.368T > C (p.F123S) was discovered and confirmed to be novel and previously unreported. Using software‐based bioinformatics analyses, we deduced that the two mutation sites are highly conserved across a wide range of species, with the ability to alter different phosphorylation sites and destabilize the ASNS protein structure. The newly identified p.F123S mutation was predicted to be the most significantly destabilizing and detrimental mutation to the ASNS protein structure, compared to all other previously reported mutations.

**Conclusion:**

Evidently, the presence of these compound heterozygous mutations could lead to severe clinical phenotypes and serve as a potential indicator for considerably higher risk with less optimistic prognosis in ASNSD patients.

## Graphical abstract

Asparagine synthetase deficiency (ASNSD) is an extremely rare pediatric congenital disorder with severe progressive microcephaly, severe global developmental delay, spastic quadriplegia, and refractory seizures as the main clinical manifestations. Mutations in asparagine synthetase (ASNS) are considered the primary culprits for ASNSD patients. Our study here has expended the current mutations spectrum and database for human ASNS with the newly identified mutation p.F123S as not only a potential risk factor for more severe phenotypes, but also a prognosis for ASNSD patients.

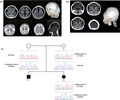

## 1 INTRODUCTION

Asparagine synthetase deficiency (ASNSD, OMIM accession number: 615574) is a rare neurometabolic disorder that was first reported by Ruzzo et al. (2013). It is clinically characterized by a triad of congenital progressive microcephaly, profound developmental delay, and axial hypotonia followed by spastic quadriplegia. Other associated clinical manifestations include micrognathia, receding forehead, large ears, intractable seizures, jitteriness, hyperekplexia, apnea, feeding difficulties, and cortical blindness. Pathologically, low asparagine levels in cerebrospinal fluid (CSF) could help differentiate this disease from other neural developmental disorders (Alfadhel et al., 2015; Ruzzo et al., 2013).

Genetically, ASNSD is an autosomal recessive disorder that can be found in newborns with either homozygous or compound heterozygous mutations in the *ASNS* (encodes asparagine synthetase) on chromosome 7q21 (Ruzzo et al., 2013). The fact that children with ASNSD are all born with microcephaly, developmental delay, and epileptic seizures suggests that ASNS protein is critical for the early development of the brain (Lomelino, Andring, McKenna, & Kilberg, 2017).

Cases of ASNSD reported from a variety of ethnic origins in the past few years (Abhyankar et al., 2018; Alfadhel et al., 2015; Ben‐Salem et al., 2015; Chen, Li, Wang, Chen, & Hong, 2019; Galada et al., 2018; Gataullina et al., 2016; Gupta et al., 2017; Palmer et al., 2015; Radha Rama Devi & Naushad, 2019; Ruzzo et al., 2013; Sacharow et al., 2018; Schleinitz et al., 2018; Seidahmed et al., 2016; Sprute et al., 2019; Sun et al., 2017; Yamamoto et al., 2017), have been summarized in Table 1 with information on nucleotide changes, amino acid changes, and the corresponding genotypes. ASNSD is considered ultrarare in China, with only one case reported in July 2019 (Chen et al., 2019). Here, we report the second study of ASNSD in China, about a novel missense mutation in the *ASNS* identified in a young Chinese girl. We also performed several bioinformatics predictions and analyses of these mutations to assess the evolutionary conservation of the mutation site across different species, predict protein phosphorylation site alterations, model wild‐type (WT) and mutant ASNS protein 3D structures, and to predict the stability of the tertiary protein structure.

**Table 1:** Summary table of all reported *ASNS* (NG_033870.2) mutations including mutations reported in this study

Publication	Family No.	Population	Patient No.	Nucleotide Change (New Variant)	Protein Change	Genotype
Ruzzo et al. (2013) (1)	1	Iranian Jewish	1	c.1084T > G(1)	p.F362V	Homozygous
	2	Iranian Jewish	2	c.1084T > G	p.F362V	Homozygous
			3			
	3	Bangladeshi	4	c.1648C > T (2)	p.R550C	Homozygous
			5			
			6			
	4	French Canadian	7	c.17C > A (3)/c.1648C > T	p.A6E/p.R550C	Compound heterozygous
			8			
			9			
Alfadhel et al.(2015) (2)	5	Saudi Arabian	10	c.1160A > G(4)	p.Y377C	Homozygous
			11			
Ben‐Salem et al. (2015) (4)	6	Emirati	12	c.1193A > C(5)	p.Y398C	Homozygous
Palmer et al.(2015) (5)	7	Chinese/ Brunei	13	c.866G > C (6)/c.1010C > T (7)	p.G289A/p.T337I	Compound heterozygous
Gataullina et al. (2016) (6)	8	NA	14	c.1439C > T (8)/c.1648 C > T	p.S480F/p.R550C	Compound heterozygous
			15			
Seidahmed et al.(2016) (7)	9	Saudi Arabian	16	c.1219C > T(9)	p.R407*	Homozygous
	10	Saudi Arabian	17	c.944A > G(10)	p.Y315C	Homozygous
Sun et al.(2017) (8)	11	Indian	18	c.1019G > A(11)	p.R340H	Homozygous
			19			
Yamamoto et al.(2017) (9)	12	Japanese	20	c.434T > C (12)/c.740T > G (13)	p.L145S/p.L247W	Compound heterozygous
	13	Japanese	21	c.1466T > A (14)/c.1623–1624del (15)	p.V489D/p.W541Cfs*	Compound heterozygous
Gupta et al.(2017) (10)	15	Indian	22	c.1138G > T (16)	p.A380S	Homozygous
Abhyankar et al.(2018) (11)	14	NA	23	c.728T > C (17)/c.1097G > A (18)	p.V243A/p.G366E	Compound heterozygous
Galada et al.(2018) (12)	16	Indian	24	c.1211G > A (19)	p.R404H	Homozygous
	17	Indian	25	c.224A > G (20)/c.413A > C (21)	p.N75S/p.D138A	Compound heterozygous
	18	Indian	26	c.1649 G > A (22)	p.R550H	Homozygous
Sacharow et al.(2018) (13)	19	Emirati	27	c.146G > A (23)	p.R49Q	Homozygous
			28			
Schleinitz et al.(2018) (14)	20	German	29	c.1165G > C (24)/c.601delA (25)	p.E389Q/p.M201Wfs*28	Compound heterozygous
			30			
Sprute et al.(2019) (15)	21	Turkish	31	c.1108C > T (26)	p.L370F	Homozygous
			32			
Radha et al.(2019) (16)	22	Indian	33	c.788C > T(27)	p.S263F	Homozygous
			34			
	23	Indian	35	c.146 G > A	p.R49Q	Homozygous
Chen et al.(2019) (17)	24	NA	36	c.1424C > T(28)/c.666_c.667delCT(29)	p.T475I/p.L222Lfs*5	Compound heterozygous
Presented case	25	Chinese	37	c.1649G > A/c.368T > C(30)	p.R550H/p.F123S	Compound heterozygous

Here, we report that one novel mutation coexists with a previously identified mutation in the *ASNS* as compound heterozygous mutations, in a Chinese girl with ASNSD. The compound heterozygous mutations not only destabilized the ASNS protein structure but also altered the phosphorylation pattern with a functionally detrimental effect on early ASNS‐correlated neural development.

## 2 MATERIALS AND METHODS

### 2.1 Participant consent and ethical compliance

The patient recruited in this study was referred to the hospital due to microcephaly and recurrent seizures. This study complied with the tenets of the Declaration of Helsinki and was approved by the Ethics Board of the Second Hospital of Dalian Medical University. Case report (CARE) guidelines were followed in this case study. A written consent from the patient and the parents was obtained prior to collecting the case material, analyzing sequencing data, and writing of the final manuscript.

### 2.2 Whole‐exome sequencing

Peripheral whole blood samples were collected from the patient in EDTA tubes, with prior consent. Genomic DNA was extracted from whole blood using the BloodGen Midi Kit (CWBIO). A DNA library containing the whole genomic DNA of the patient was prepared in an exome‐targeted/enriched/captured fashion using the xGen® Exome Research Panel v1.0 Lockdown® Probe (Integrated DNA Technologies, USA), according to the manufacturer's protocol. Whole‐exome sequencing (WES) of the enriched protein‐encoding exome was performed on an Illumina HiSeq XTen (Illumina, Inc.) platform based on the standard manufacturer guidelines. Per‐cycle BCL basecall files generated directly from Illumina HiSeq XTen were converted to per‐read FASTQ files using the Bcl2Fastq software (Illumina, Inc.) for later analysis and sequence alignment. The patient's sequence was aligned with the GRCh37 (hg19) human genome sequence using the BWA‐aligner software (version 0.6.2). Screening and annotation of SNPs and indels were performed using SAMtools (Samtools, UK) and Pindel (Sanger Institute), respectively. False‐positive variant filtration was performed based on the sequencing coverage and mutation qualities. The GenBank reference sequence and version number for *ASNS* is NG_033870.2.

### 2.3 Sanger sequencing

Sanger sequencing was performed on the peripheral blood samples taken from the patient's parents using an ABI 3730XL DNA sequencer (Applied Biosystems, USA) to verify the familial de novo inheritance of the compound mutations detected in the patient.

### 2.4 Multiple‐sequence alignment

ASNS protein sequence alignment between orthologs was performed using MEGA software (version 7) (Kumar, Stecher, & Tamura, 2016), using the ClustalW algorithm as default settings. Protein sequences retrieved from the NCBI Protein database were used in the alignment as reference sequences.

### 2.5 Protein phosphorylation site prediction

The predicted phosphorylation sites were evaluated using the web‐based server NetPhos 3.1a (Blom, Gammeltoft, & Brunak, 1999). Only those predicted phosphorylation sites with a score higher than the threshold (0.5) were included in the result.

### 2.6 Protein modeling

The ASNS protein was modeled using the I‐Tasser online server (Yang & Zhang, 2015). FASTA‐formatted WT, p.F123S, and p.R550H ASNS protein sequences were uploaded and modeled with the crystal structure from E. coli as a reference (PDB#: 1CT9), as reported by Yamamoto et al. (2017). Protein models with the top 3 scores were chosen and visualized using PyMOL software (The PyMOL Molecular Graphics System, Version 2.3.2, Schrödinger, LLC).

### 2.7 Protein stability prediction

Protein stability for all the mutant ASNS proteins was evaluated using the web‐based tools DUET (Pires, Ascher, & Blundell, 2014), INPS‐MD (Savojardo, Fariselli, Martelli, & Casadio, 2016), and MUPro (Cheng, Randall, & Baldi, 2005). ΔΔG values were recorded and visualized as the stability score by R software (version 3.6.1, The R Foundation for Statistical Computing Platform) using “pheatmap” package (Kolde, 2012).

## 3 RESULTS

### 3.1 Clinical presentation

**Figure 1:**
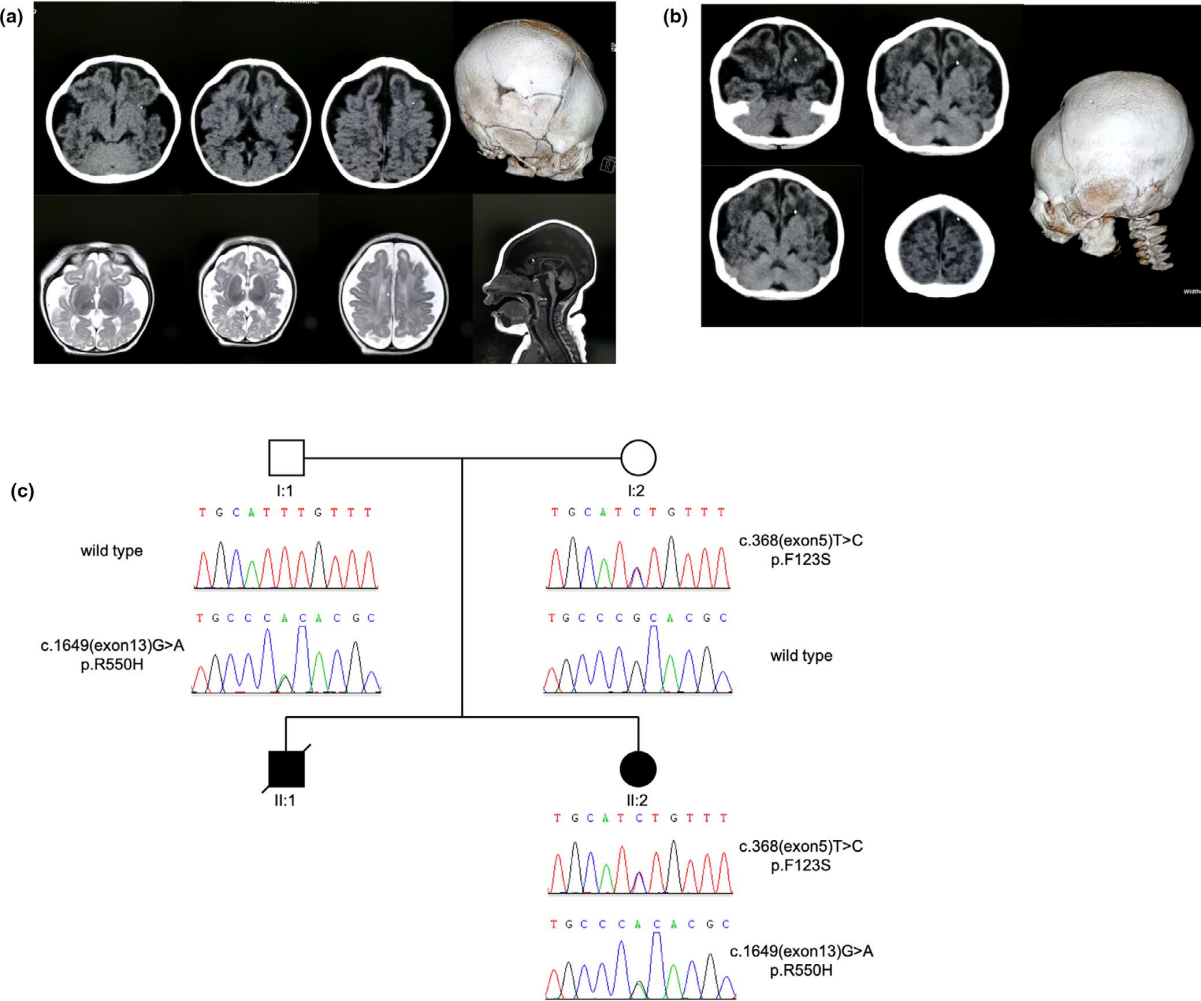
Clinical and genetic diagnosis of ASNSD in the patients. (a) Brain Computerized Tomography (CT) scan and magnetic resonance imaging (MRI) for the patient at the age of 2 months, showing clinical onset of severe microcephaly. (b) CT scan for the patient’s older brother at the age of 4 months, showing similar image pattern as the patient. (c) Pedigree analysis of patient’s family. Underneath each family member are the corresponding genotypes and sanger sequencing results, showing mutation c.368(exon 5)T>C was maternally inherited and c.1649(exon 13)G>A was paternally inherited. II:1 is the patient’s brother who unfortunately passed away. II:2 is the patient herself with mutation acquired from both parents

The patient was born through spontaneous vaginal delivery as the second child of a nonconsanguineous Chinese couple with no family history of genetic disorders. Her older brother died 4 months after birth due to microcephaly and feeding difficulty. The brain computerized tomography (CT) scan images of the older brother (shown in Figure 1b) exhibited distinct microcephaly compared to the brain of an average Chinese male infant. Genetic tests were not conducted for the patient's deceased older brother due to late admission to the hospital.

At the age of 2 months, the female patient in our study visited the outpatient department of our hospital complaining of microcephaly and impaired response to visual stimuli. At 3 months, the patient started to develop recurrent seizures manifested as rapid blinking, flexing, and shaking with loss of consciousness in both upper limbs, occurring a dozen times per day for a duration of approximately 1 min per attack. Physical examination revealed that the patient was suffering from microcephaly with a head circumference (HC) of 35.5 cm (mean: 3SD 36.0 cm, based on the HC of average Chinese female children at 3 months of age). In addition, the patient had never acquired due developmental milestones. Such developmental retardation was also concluded by weakened head‐controlling ability, visual pursuit ability, and aural stimuli responding ability. Severe hypermyotonia in all four limbs and severe hyperfunction of tendon reflexes were also detected.

Despite all the above physical examinations, the results of the brain CT scan and magnetic resonance imaging (MRI) revealed onsets of microcephaly, similar to the patient's older brother (Figure 1b), specifically with a flat frontal skull, reduced cranial cavity, decreased cerebral volume, simplified gyral pattern, leukoaraiosis, ventriculomegaly, and enlarged axial spaces (Figure 1a.

To unravel the origin of the seizure, 2 hr of video electroencephalogram (EEG) was recorded for the patient. The EEG recording indicated multifocal epileptiform discharges in the bilateral frontal, frontal, and middle line regions. Moreover, the epileptiform discharges were shown to particularly concentrate on the right side (Figure S1). The patient was subsequently administered levetiracetam, which transiently ameliorated the frequency and severity of the seizures. However, the patient's parents failed to regularly administer an antiepileptic drug as advised, and her seizures continued to exacerbate.

As for standard laboratory examinations including urine and blood tests, the results did not show any abnormality in terms of urine organic acids, blood amino acids, and acylcarnitine.

Based on these clinical features of congenital microcephaly, global developmental delay, axial hypotonia, hypermyotonia of four limbs, seizures, and generalized brain atrophy as well as her family history, we tended to diagnose the condition to be ASNSD. Genetic tests were performed on this patient after the clinical diagnosis of ASNSD to reveal the molecular etiology.

### 3.2 Genomic analysis and pedigree analysis of the patient and the parents

WES was performed on the committed patient. As a result, heterozygous compound mutations in *ASNS* were revealed*.* Standard Sanger sequencing was performed on DNA isolated from whole blood of the parents. It was confirmed that the compound mutations in *ASNS* consist of two missense mutations, c.368T > C (maternally inherited) and c.1649G > A (paternally inherited), which subsequently lead to amino acid sequence modifications of p.F123S (amino acid phenylalanine to serine) and p.R550H (amino acid arginine to histamine), respectively. The paternally acquired c.1649G > A mutation was previously reported in India in the form of a homozygous mutation (Galada et al., 2018), with a minor allele frequency (MAF) of 0.0002. However, c.368T > C was not found in any open variant databases, including gnomAD, ClinVar, and ExAC. Hence, c.368T > C has been identified and reported by us for the first time as a novel mutation.

To better visualize the inheritance relationship between the patient and the parents, a pedigree tree was constructed for the patient's family (Figure 1c). As shown in Figure 1c, the paternally inherited c.1649G > A and maternally inherited c.368T > C were passed down to the patient and are responsible for the onset of ASNSD.

### 3.3 Overview of *ASNS* and ASNS protein conservation across species

**Figure 2:**
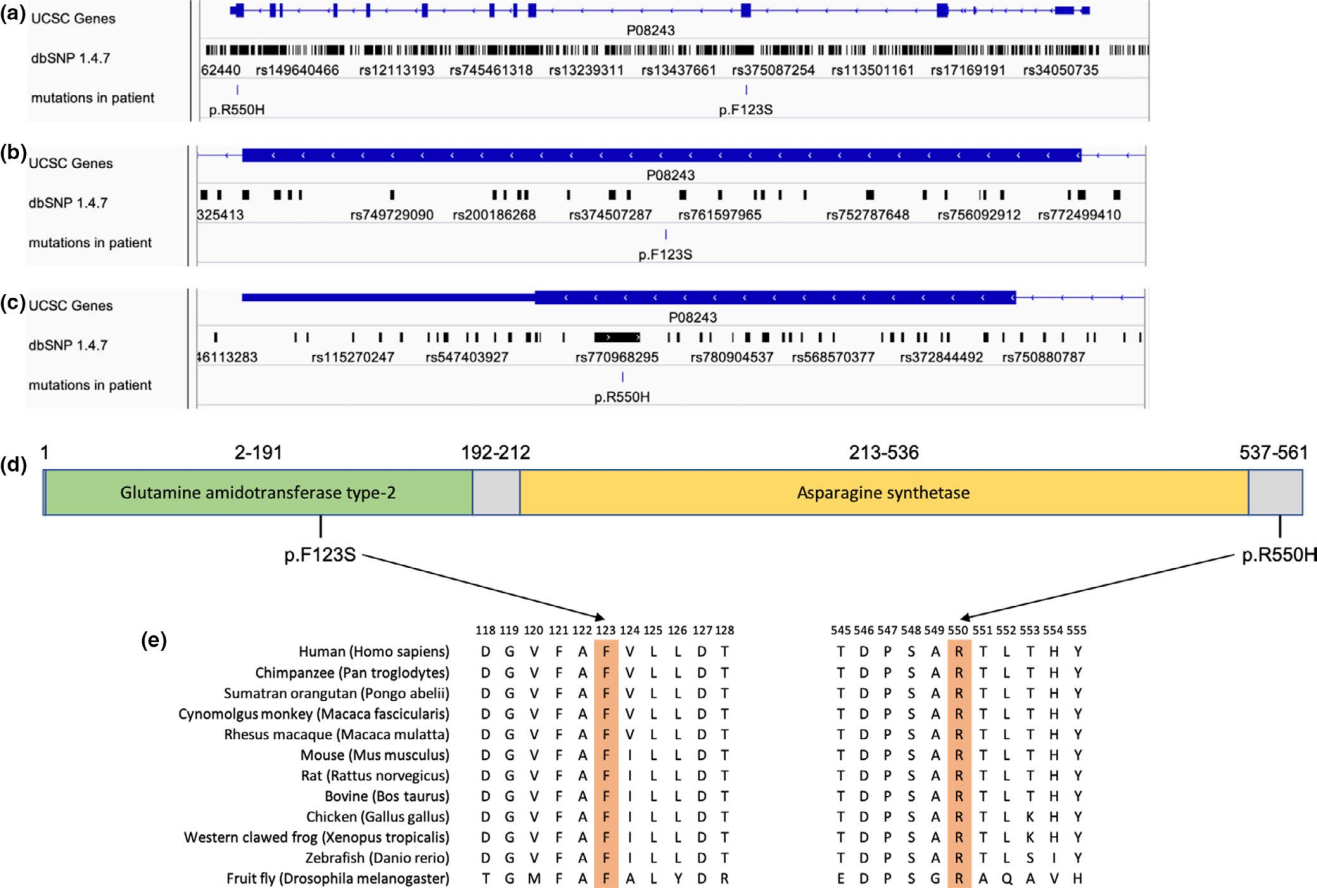
Overview and visualization of *ASNS* (NG_033870.2) gene and the identified mutations. (a) IGV genome browser overview for *ASNS* gene showing both mutations sites simultaneously. (b and c) Zoomed in view of the identified mutations from the patient showing both mutations reside in exon area. The mutation p.F123S does not overlap with any mutations in dbSNP 1.4.7 database. (d) 2 major functional domains within ASNS protein based on UniProt. Green domain being the glutamine amidotransferase type‐2 and the orange being the asparagine synthetase. (e) Conservation analysis of ASNS protein sequences across different species. Amino acid positions of both mutations are highlighted in orange. All the amino acids sequences were retrieved form NCBI Protein database: NP_001339425.1 (*Homo sapiens*); NP_001267331.1 (*Pan troglodytes*); NP_001126469.1 (*Pongo abelii*); XP_015303200.1 (*Macaca fascicularis*); XP_028701873.1 (*Macaca mulatta*); NP_036185.1 (*Mus musculus*); NP_037211.2 (*Rattus norvegicus*); NP_001069121.1 (*Bos Taurus*); NP_001026148.1 (*Gallus gallus*); NP_001265452.1 (*Xenopus tropicalis*); NP_957457.3 (*Danio rerio*); NP_996132.1 (*Drosophila melanogaster*). Amino acids phenylalanine at position 123 and arginine at position 550 are highly conserved through these species

To better review the location of the mutations within the ASNS gene, we used the IGV genome browser (Robinson et al., 2011) to visualize the two mutation sites (Figure 2a). It is revealed that both mutations located in the exon area (Figures 2b and c).

At the protein level, based on the description retrieved from UniProt (UniProt, 2019), the ASNS protein contains two functional domains named glutamine amidotransferase type‐2 domain (amino acid 2–191, Figure 2d) and asparagine synthetase domain (amino acids 213–536, Figure 2d). The mutation p.F123S was shown to be located in the glutamine amidotransferase type‐2 domain (Figure 2e), while p.R550H resides in the C‐terminal of the ASNS protein sequence, rather than in either of the functional domains (Figure 2e). To test the evolutionary conservation of the two amino acids in WT ASNS, we compared wild‐type amino acid sequences of ASNS across 12 different species, including *Homo sapiens*, nonhuman primates, rodents, artiodactyla, aves, amphibians, fish, and arthropods. The results demonstrated that both the amino acids were reported to be highly conserved across all 12 species, suggesting that both amino acids play crucial roles in ASNS in the course of evolution and the maintenance of biochemically relevant activities (Figure 2e). The results of the conservation analysis can be found in Table 1. It is expected that the phenylalanine at amino acid position 123 is functionally essential because of its location, whereas the arginine at position 550 was unexpectedly conserved across all 12 species. Consequently, it is suspected that the arginine at amino acid position 550 may participate in the construction of the protein tertiary structure, which partially dictates ASNS functional performance.

### 3.4 ASNS phosphorylation site prediction

**Figure 3:**
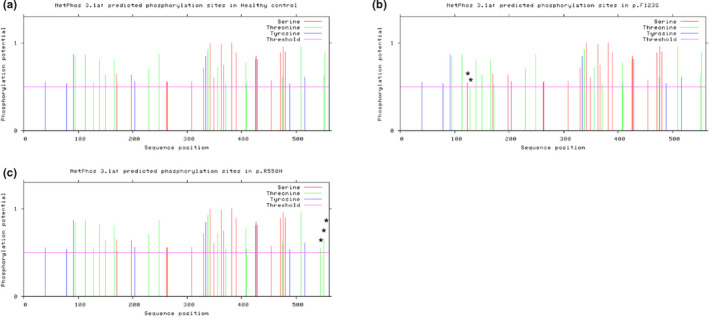
Mutations altered phosphorylation sites of ASNS protein predicted by NetPhos 3.1 Server. All phosphorylation sites with phosphorylation potential score (PPS) over the threshold (PPS = 0.5) were recorded in the figure. (a–c) The predicted phosphorylation sites for healthy control, p.F123S and p.R550H respectively. “*” indicates the changes of phosphorylation sites caused by p.F123S and p.R550H compared to healthy control

To further study the potential functional impact of the mutations on ASNS protein, phosphorylation sites for serine, threonine, and tyrosine as well as their responsible kinases were predicted and assessed using NetPhos 3.1 Server (Blom et al., 1999; Blom, Sicheritz‐Ponten, Gupta, Gammeltoft, & Brunak, 2004). In total, 44 serine, threonine, and tyrosine phosphorylation sites that have significant phosphorylation potential were reported in healthy controls (accession: NP_001339425) (Figure 3a), 45 phosphorylation sites were predicted in the p.F123S variant, with the mutant serine amino acid as the newly acquired phosphorylation site (PKA is the predicted responsible kinase) (Figure 3b). In addition to the serine phosphorylation site, the downstream phosphorylation site at amino acid position 128 (threonine) was also affected by p.F123S. The phosphorylation potential score (PPS) was reduced from 0.548 to 0.501 (0.5 is the significance threshold, Table. 2).

**Table 2:** Altered phosphorylation sites in mutant form of ASNS protein as reported in Fig. 3 with PPS as well as the corresponding kinases

Mutation	Amino acid	Sample	Context	Score	Kinase
p.F123S	123 F/S	Healthy control	NA		
		Patient	GVFASVLLD	0.542	PKA
	128 T	Healthy control	VLLDTANKK	0.548	PKC
		Patient	VLLDTANKK	0.501	PKC
P.R550H	545 T	Healthy control	NA		
		Patient	WINATDPSA	0.549	PKC
	551 T	Healthy control	PSARTLTHY	0.629	PKC
		Patient	PSAHTLTHY	0.652	PKC
	553 T	Healthy control	ARTLTHYKS	0.886/0.510	unsp/RSK
		Patient	NA		

The total number of phosphorylation sites in the p.R550H mutant variant was 44 (Figure 3c), which is consistent with the healthy control. However, there were three major changes. First, the threonine at the 545 amino acid position in the healthy control was not originally a phosphorylation site. However, the p.R550H mutation enhanced its phosphorylation potential and turned it into a phosphorylation site with PKC as the kinase candidate (PPS = 0.549, Table. 2). Second, the p.R550H mutant also slightly increased the PPS of the threonine at 551 amino acid position from 0.629 to 0.652 (Table. 2). Third, in WT ASNS, the threonine at 553 amino acid position could be either strongly phosphorylated by an unspecified kinase (unsp, PPS = 0.886) or weakly by RSK (PPS = 0.510); however, the p.R550H mutation attenuated this phosphorylation site.

In general, both mutations have the ability to alter the phosphorylation potential of several different amino acid sites within the protein sequence, suggesting that these two mutations predictably influence the biological functions of ASNS by changing its phosphorylation status. Such fundamental in silico protein analysis still requires further confirmation via functional experimental approaches.

### 3.5 ASNS protein structure 3D modeling

**Figure 4:**
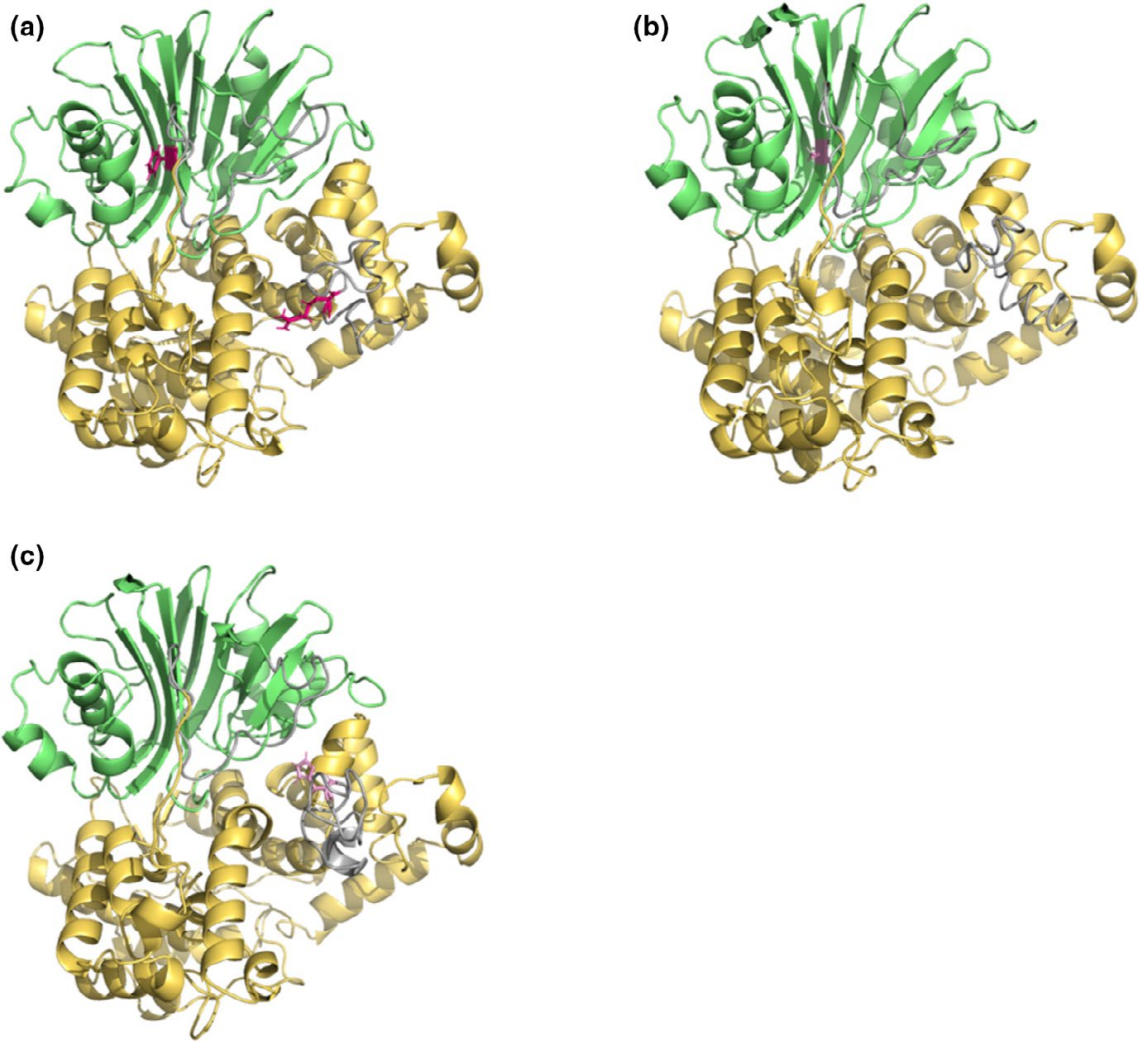
Protein structure 3D modeling. (a–c) 3D models for healthy control, p.F123S and p.R550H mutant types respectively. Glutamine amidotransferase type‐2 domain and asparagine synthetase domain are highlighted in green and yellow respectively. The original amino acids in normal ASNS protein at position 123 and 550 are marked in red in a and the mutations are marked pink in b and c respectively

Protein structure 3D modeling was performed using I‐TASSER Server (Yang & Zhang, 2015; Zhang, Freddolino, & Zhang, 2017), with a previously established crystalized ASNS protein structure from E. coli as the reference model (PDB#: 1CT9). As we described above, ASNS has a glutamine amidotransferase type‐2 domain and a asparagine synthetase domain, which are represented by green and yellow colors, respectively, (Figure 4a). The nonfunctional regions are marked in gray (Figure 4a). Amino acids in the WT ASNS at positions 123 and 550 are shown in red in Figure 4a, and the mutant amino acids are highlighted in pink in Figure 4b and c. To analyze the downstream effects of the two mutations on ASNS protein structure stability, DUET (Pires et al., 2014), INPS‐MD (Savojardo et al., 2016), and MUPro (Cheng et al., 2005) were employed. It was shown that both missense mutations had a damaging effect on the ASNS protein structure stability (Figure S2), with p.F123S being the most destabilizing mutation to the ASNS protein structure among all the previously reported mutations. The ΔΔG values for p.F123S based on all three prediction tools were less than −2, which further indicated the detrimental influence on the stability of the ASNS protein exerted by p.F123S, thus damaging the biological functions of ASNS. Moreover, mutation p.R550H showed a mild damaging effect on ASNS protein stability, as judged by its closer‐to‐zero ΔΔG values (0.181793, −0.7, −1.324, respectively, in INPS‐MD, DUET, and MUPro (Figure S2)).

## 4 DISCUSSION AND CONCLUSIONS

ASNSD is a rare congenital disorder that is clinically manifested by microcephaly, global developmental delay, spastic quadriplegia, and refractory seizures. Exome sequencing has been widely applied to identify the genetic etiology of all 37 previously reported ASNSD cases in 25 unrelated families since 2013. Hitherto, 30 disease‐causing mutant variants in the *ASNS* have been identified, most of which were due to recessive missense mutations (Table. 1).

Based on these reported mutations, the underlying mechanism behind the clinical manifestation of ASNSD is entirely driven by either compromised protein function or partial protein truncation due to premature stop codons introduced by mutations. Severe disease variants not only comprised of homozygous mutants but also compound heterozygous mutations conjunctly inherited from both parents. The homozygous to compound heterozygous reported case ratio was 3:2.

Before the patient in our case study was committed to the hospital due to microcephaly, her older brother was also diagnosed with microcephaly and passed away due to feeding difficulties together with respiratory functional attenuation. These are the two main causes of early infancy death in congenital cerebral development‐related disorders (Ruzzo et al., 2013). Considering the recurrence of microcephalic newborns within a single family and the typical clinical manifestations of abnormally small HC, severely delayed development, and hypermyotonia, the patient exhibited adequate evidence of inheritable developmental disorder (Figure 1c). Therefore, WES was applied to screen for the responsible gene, and diagnosis was soon established as ASNSD, caused by heterozygous compound mutations in the *ASNS*.

The production of asparagine from aspartate via an amidation reaction in the presence of ATP, coupled with glutamate and ammonia yielded from its N‐terminal, is the primary mechanism of action and function of ASNS. Hence, losing ASNS will result in an apparent deficiency in cellular asparagine levels (Richards & Kilberg, 2006). However, our patient's plasma and urine asparagine levels were within the normal range, which is likely related to nonmedical conditions such as food intake and daily activities, as reported previously (Van Der Crabben et al., 2013).

It has been demonstrated that asparagine and ASNS are critical for both embryonic and postembryonic early neurological and cerebral development (Ruzzo et al., 2013; Scholl‐Burgi et al., 2008). The MRI results of our patient matched with the classic MRI pattern for ASNSD, reduced cranial cavity, simplified gyral pattern, cerebral atrophy, leukoaraiosis, and cerebral ventriculomegaly (Figure 1a).

In our study, two missense mutations were identified as compound heterozygous mutations in our ASNSD patient (Figure 1c). One of the mutations c.1649G > A (p.R550H) has been previously reported in India by Galada et al. without in‐depth study and software‐based analysis to deduce its downstream biological functions (Galada et al., 2018). The other mutation c.368T > C (p.F123S), however, is reported for the first time.

Phosphorylation site alterations impacted by the two mutations were tested and predicted using the NetPhos 3.1 Server (Blom et al., 1999, 2004). The most impactful phosphorylation site alterations are the loss of phosphorylation site on 553T with RSK as the operating kinase due to the p.R550H mutation, the gain of phosphorylation site on 545T amino acid position with PKC as the responsible kinase due to the p.R550H mutation and the gain of phosphorylation site on 123S with PKA as the kinase responsible for p.F123S mutation. Other minor phosphorylation site modifications were also predicted on 128T (decrease phosphorylation potential) and 551T (increase phosphorylation potential). However, such an in silico phosphorylation site prediction is not powerful enough to conclude its impacts on downstream biological functions. In future, functional studies and in situ experimental approaches are needed to investigate their impacts on ASNS enzymatic activity, ATP‐binding potential, GATase activity, and glutamate and ammonia conversion from the N‐terminal.

Despite the phosphorylation site prediction, web tool‐based protein stability prediction was also performed on all previously identified amino acid mutations, including the two mutations identified in our patient. According to INPS, DUET, and MUPro, the newly identified mutation p.F123S is the most destabilizing mutation among all other mutations. As a result, it is predicted that the majority of the ASNS protein expressed during the patient's early embryonic development would be either poorly functional or completely attenuated. To some degree, the patient will eventually encounter feeding, pulmonary, and cardiovascular challenges, leading to neonatal death. Previously, a different variant was reported on the amino acid position 550 with a substituted amino acid of cysteine replacing arginine (p.R550C) (Ruzzo et al., 2013). The downstream influence on the protein expression level of the p.R550C variant was investigated by transfecting COS‐7 cells (Monkey kidney fibroblast‐like cell line) with a vector containing the p.R550C variant *ASNS* sequence. It was shown that the p.R550C variant resulted in an upregulation of ASNS protein abundance (Ruzzo et al., 2013). In future, similar functional examinations should be performed on novel mutations.

To the best of our knowledge, there are no effective therapies for ASNSD, let alone a functional cure. Most of the ASNSD cases were discovered postnatally with severe onset of microcephaly among other developmental abnormalities. By the time ASNSD was diagnosed, irreversible damages have already been exerted prenatally. Therefore, all existing treatments target supportive care and external administration of asparagine. However, it was previously reported that oral or intravenous administration of asparagine might not be effective for the elevation of cerebral asparagine levels due to its delivery kinetics (Palmer et al., 2015). Risk disclosure of ASNSD, however, is applicable by applying prenatal genetic tests on the fetus and prepregnancy genetic tests of the parents. In our opinion, the most effective therapeutic intervention that can achieve functional cure relies on gene manipulation‐based therapy.

## CONFLICT OF INTEREST

The authors declare they have no conflict of interest.

## AUTHORS CONTRIBUTION

Li Z and Lin Y designed and supervised this study. Wang C and He G presented the clinical information of the patient and performed the following bioinformatic analyses. Li R provided the clinical consultation for the patient. Wang C, He G, and Ge Y prepared the manuscript. All authors provided their feedbacks on the case presentation, bioinformatic analyses, and the final manuscript.

## Supporting information

Fig S1

Fig S2

Table S1

## References

[mgg31235-bib-0001] Abhyankar, A. , Lamendola‐Essel, M. , Brennan, K. , Giordano, J. L. , Esteves, C. , Felice, V. , … Jobanputra, V. (2018). Clinical whole exome sequencing from dried blood spot identifies novel genetic defect underlying asparagine synthetase deficiency. Clinical Case Reports, 6(1), 200–205. 10.1002/ccr3.1284 29375865 PMC5771929

[mgg31235-bib-0002] Alfadhel, M. , Alrifai, M. T. , Trujillano, D. , Alshaalan, H. , Al Othaim, A. , Al Rasheed, S. , … Eyaid, W. (2015). Asparagine synthetase deficiency: New inborn errors of metabolism. JIMD Reports, 22, 11–16. 10.1007/8904_2014_405 25663424 PMC4486270

[mgg31235-bib-0003] Ben‐Salem, S. , Gleeson, J. G. , Al‐Shamsi, A. M. , Islam, B. , Hertecant, J. , Ali, B. R. , & Al‐Gazali, L. (2015). Asparagine synthetase deficiency detected by whole exome sequencing causes congenital microcephaly, epileptic encephalopathy and psychomotor delay. Metabolic Brain Disease, 30(3), 687–694. 10.1007/s11011-014-9618-0 25227173 PMC4915861

[mgg31235-bib-0004] Blom, N. , Gammeltoft, S. , & Brunak, S. (1999). Sequence and structure‐based prediction of eukaryotic protein phosphorylation sites. Journal of Molecular Biology, 294(5), 1351–1362. 10.1006/jmbi.1999.3310 10600390

[mgg31235-bib-0005] Blom, N. , Sicheritz‐Ponten, T. , Gupta, R. , Gammeltoft, S. , & Brunak, S. (2004). Prediction of post‐translational glycosylation and phosphorylation of proteins from the amino acid sequence. Proteomics, 4(6), 1633–1649. 10.1002/pmic.200300771 15174133

[mgg31235-bib-0006] Chen, B. , Li, W. , Wang, X. , Chen, K. , & Hong, X. (2019). Cyst‐peritoneal shunt for the treatment of a progressive intracerebral cyst associated with ASNS mutation: Case report and literature review. World Neurosurgery, 127, 1–7. 10.1016/j.wneu.2019.02.130 30844524

[mgg31235-bib-0007] Cheng, J. , Randall, A. , & Baldi, P. (2005). Prediction of protein stability changes for single‐site mutations using support vector machines. Proteins: Structure, Function, and Bioinformatics, 62(4), 1125–1132. 10.1002/prot.20810 16372356

[mgg31235-bib-0008] Galada, C. , Hebbar, M. , Lewis, L. , Soans, S. , Kadavigere, R. , Srivastava, A. , … Shukla, A. (2018). Report of four novel variants in ASNS causing asparagine synthetase deficiency and review of literature. Congenital Anomalies, 58(5), 181–182. 10.1111/cga.12275 29405484 PMC6338226

[mgg31235-bib-0009] Gataullina, S. , Lauer‐Zillhardt, J. , Kaminska, A. , Galmiche‐Rolland, L. , Bahi‐Buisson, N. , Pontoizeau, C. , … Fallet‐Bianco, C. (2016). Epileptic phenotype of two siblings with asparagine synthesis deficiency mimics neonatal pyridoxine‐dependent epilepsy. Neuropediatrics, 47(6), 399–403. 10.1055/s-0036-1586222 27522229

[mgg31235-bib-0010] Gupta, N. , Tewari, V. V. , Kumar, M. , Langeh, N. , Gupta, A. , Mishra, P. , … Kabra, M. (2017). Asparagine Synthetase deficiency‐report of a novel mutation and review of literature. Metabolic Brain Disease, 32(6), 1889–1900. 10.1007/s11011-017-0073-6 28776279

[mgg31235-bib-0011] Kolde, R. (2012). Pheatmap: Pretty heatmaps. R Package Version, 61(926), 915.

[mgg31235-bib-0012] Kumar, S. , Stecher, G. , & Tamura, K. (2016). MEGA7: Molecular evolutionary genetics analysis version 7.0 for bigger datasets. Molecular Biology and Evolution, 33(7), 1870–1874. 10.1093/molbev/msw054 27004904 PMC8210823

[mgg31235-bib-0013] Lomelino, C. L. , Andring, J. T. , McKenna, R. , & Kilberg, M. S. (2017). Asparagine synthetase: Function, structure, and role in disease. Journal of Biological Chemistry, 292(49), 19952–19958. 10.1074/jbc.R117.819060 29084849 PMC5723983

[mgg31235-bib-0014] Palmer, E. E. , Hayner, J. , Sachdev, R. , Cardamone, M. , Kandula, T. , Morris, P. , … Kirk, E. P. (2015). Asparagine Synthetase Deficiency causes reduced proliferation of cells under conditions of limited asparagine. Molecular Genetics and Metabolism, 116(3), 178–186. 10.1016/j.ymgme.2015.08.007 26318253 PMC10152381

[mgg31235-bib-0015] Pires, D. E. V. , Ascher, D. B. , & Blundell, T. L. (2014). DUET: A server for predicting effects of mutations on protein stability using an integrated computational approach. Nucleic Acids Research, 42(W1), W314–W319. 10.1093/nar/gku411 24829462 PMC4086143

[mgg31235-bib-0016] Radha Rama Devi, A. , & Naushad, S. M. . (2019). Molecular diagnosis of asparagine synthetase (ASNS) deficiency in two Indian families and literature review of 29 ASNS deficient cases. Gene, 704, 97–102. 10.1016/j.gene.2019.04.024 30978478

[mgg31235-bib-0017] Richards, N. G. J. , & Kilberg, M. S. (2006). Asparagine synthetase chemotherapy. Annual Review of Biochemistry, 75(1), 629–654. 10.1146/annurev.biochem.75.103004.142520 PMC3587692 16756505

[mgg31235-bib-0018] Robinson, J. T. , Thorvaldsdottir, H. , Winckler, W. , Guttman, M. , Lander, E. S. , Getz, G. , & Mesirov, J. P. (2011). Integrative genomics viewer. Nature Biotechnology, 29(1), 24–26. 10.1038/nbt.1754 PMC3346182 21221095

[mgg31235-bib-0019] Ruzzo, E. K. , Capo‐Chichi, J.‐M. , Ben‐Zeev, B. , Chitayat, D. , Mao, H. , Pappas, A. L. , … Goldstein, D. B. (2013). Deficiency of asparagine synthetase causes congenital microcephaly and a progressive form of encephalopathy. Neuron, 80(2), 429–441. 10.1016/j.neuron.2013.08.013 24139043 PMC3820368

[mgg31235-bib-0020] Sacharow, S. J. , Dudenhausen, E. E. , Lomelino, C. L. , Rodan, L. , El Achkar, C. M. , Olson, H. E. , … Kilberg, M. S. (2018). Characterization of a novel variant in siblings with Asparagine Synthetase Deficiency. Molecular Genetics and Metabolism, 123(3), 317–325. 10.1016/j.ymgme.2017.12.433 29279279 PMC5832599

[mgg31235-bib-0021] Savojardo, C. , Fariselli, P. , Martelli, P. L. , & Casadio, R. (2016). INPS‐MD: A web server to predict stability of protein variants from sequence and structure. Bioinformatics, 32(16), 2542–2544. 10.1093/bioinformatics/btw192 27153629

[mgg31235-bib-0022] Schleinitz, D. , Seidel, A. , Stassart, R. , Klammt, J. , Hirrlinger, P. G. , Winkler, U. , … Hirrlinger, J. (2018). Novel mutations in the asparagine synthetase gene (ASNS) associated with microcephaly. Frontiers in Genetics, 9, 245 10.3389/fgene.2018.00245 30057589 PMC6053511

[mgg31235-bib-0023] Scholl‐Burgi, S. , Haberlandt, E. , Heinz‐Erian, P. , Deisenhammer, F. , Albrecht, U. , Sigl, S. B. , … Karall, D. (2008). Amino acid cerebrospinal fluid/plasma ratios in children: Influence of age, gender, and antiepileptic medication. Pediatrics, 121(4), e920–e926. 10.1542/peds.2007-1631 18332074

[mgg31235-bib-0024] Seidahmed, M. Z. , Salih, M. A. , Abdulbasit, O. B. , Samadi, A. , Al Hussien, K. , Miqdad, A. M. , … Alkuraya, F. S. (2016). Hyperekplexia, microcephaly and simplified gyral pattern caused by novel ASNS mutations, case report. BMC Neurology, 16, 105 10.1186/s12883-016-0633-0 27422383 PMC4947274

[mgg31235-bib-0025] Sprute, R. , Ardicli, D. , Oguz, K. K. , Malenica‐Mandel, A. , Daimagüler, H.‐S. , Koy, A. , … Cirak, S. (2019). Clinical outcomes of two patients with a novel pathogenic variant in ASNS: Response to asparagine supplementation and review of the literature. Human Genome Variation, 6, 24 10.1038/s41439-019-0055-9 31123592 PMC6531480

[mgg31235-bib-0026] Sun, J. , McGillivray, A. J. , Pinner, J. , Yan, Z. , Liu, F. , Bratkovic, D. , … Chopra, M. (2017). Diaphragmatic eventration in sisters with asparagine synthetase deficiency: A novel homozygous ASNS mutation and expanded phenotype. JIMD Reports, 34, 1–9. 10.1007/8904_2016_3 27469131 PMC5509547

[mgg31235-bib-0027] UniProt, C. (2019). UniProt: A worldwide hub of protein knowledge. Nucleic Acids Research, 47(D1), D506–D515. 10.1093/nar/gky1049 30395287 PMC6323992

[mgg31235-bib-0028] Van Der Crabben, S. N. , Verhoeven‐Duif, N. M. , Brilstra, E. H. , Van Maldergem, L. , Coskun, T. , Rubio‐Gozalbo, E. , … De Koning, T. J. (2013). An update on serine deficiency disorders. Journal of Inherited Metabolic Disease, 36(4), 613–619. 10.1007/s10545-013-9592-4 23463425

[mgg31235-bib-0029] Yamamoto, T. , Endo, W. , Ohnishi, H. , Kubota, K. , Kawamoto, N. , Inui, T. , … Fukao, T. (2017). The first report of Japanese patients with asparagine synthetase deficiency. Brain and Development, 39(3), 236–242. 10.1016/j.braindev.2016.09.010 27743885

[mgg31235-bib-0030] Yang, J. , & Zhang, Y. (2015). I‐TASSER server: New development for protein structure and function predictions. Nucleic Acids Research, 43(W1), W174–W181. 10.1093/nar/gkv342 25883148 PMC4489253

[mgg31235-bib-0031] Zhang, C. , Freddolino, P. L. , & Zhang, Y. (2017). COFACTOR: Improved protein function prediction by combining structure, sequence and protein‐protein interaction information. Nucleic Acids Research, 45(W1), W291–W299. 10.1093/nar/gkx366 28472402 PMC5793808

