# Supplementary figures and images for: A novel compound heterozygous missense mutation in ASNS broadens the spectrum of asparagine synthetase deficiency
Source: Mol Genet Genomic Med. 2020 Apr 7;8(6):e1235. doi: 10.1002/mgg3.1235 (PMC7284041; doi:10.1002/mgg3.1235)

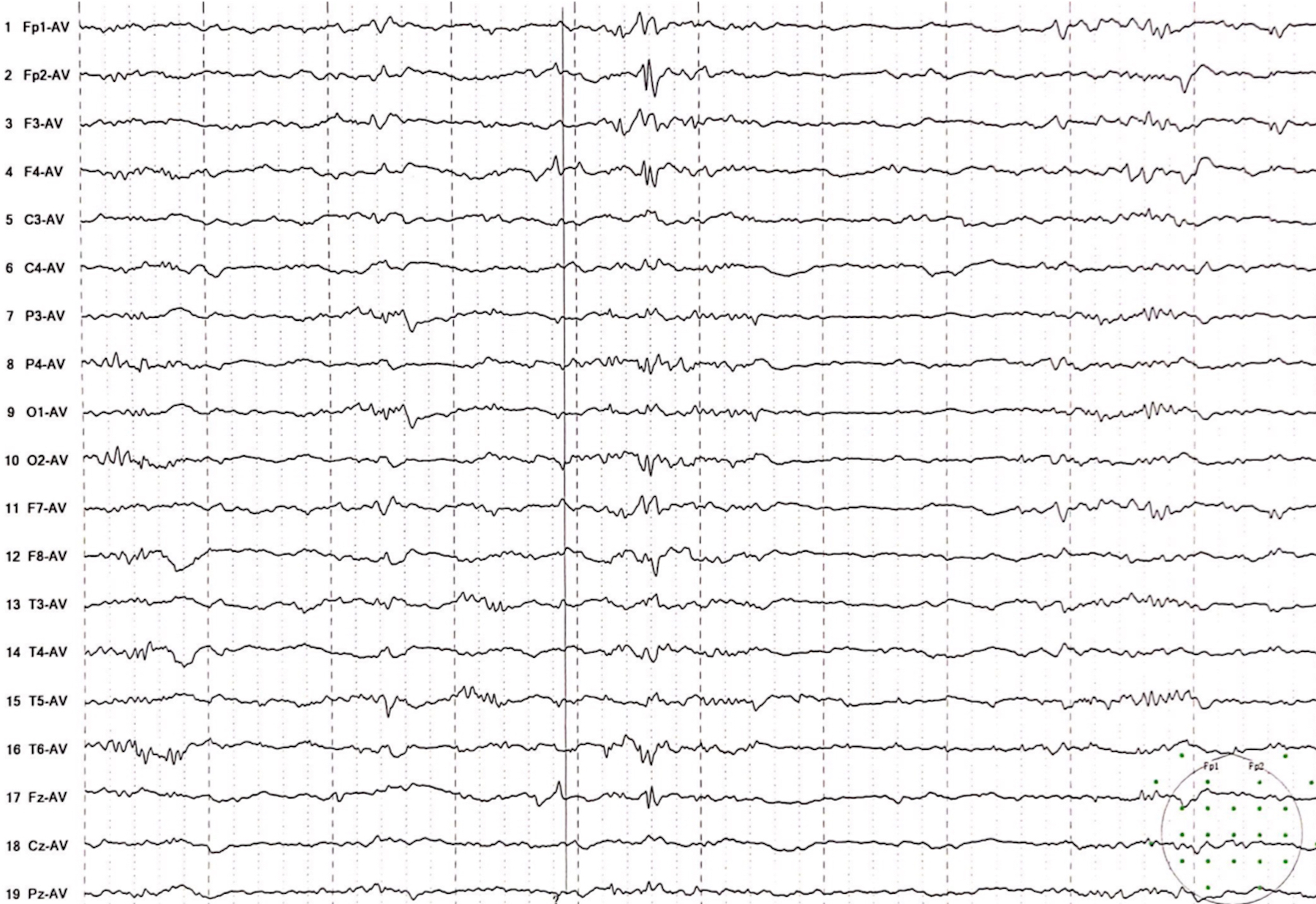

Supplement: Supplementary file 1 — Fig S1 [file MGG3-8-e1235-s001.jpg]

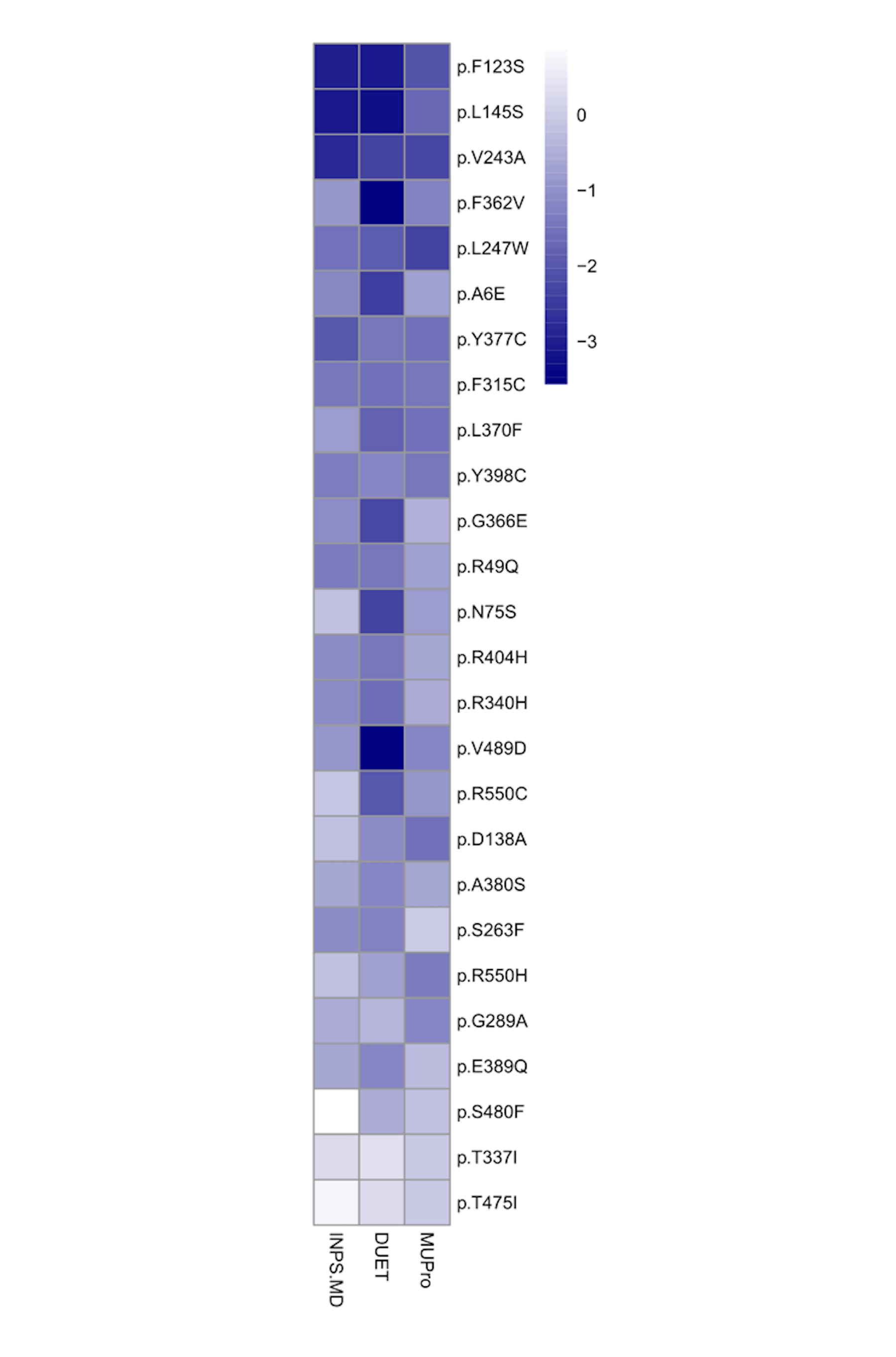

Supplement: Supplementary file 2 — Fig S2 [file MGG3-8-e1235-s002.jpg]
