# Supplementary material for: A novel compound heterozygous missense mutation in ASNS broadens the spectrum of asparagine synthetase deficiency
Source: Mol Genet Genomic Med. 2020 Apr 7;8(6):e1235. doi: 10.1002/mgg3.1235 (PMC7284041; doi:10.1002/mgg3.1235)
Supplement: Supplementary file 3 — Table S1 [file MGG3-8-e1235-s003.docx]

| Human (Homo sapiens) | M | C | G | I | W | A | L | F | G | S | D | - | - | - | - | - | - | D | C | L | S | V | Q | C | L | S | A | M | K | I | A | H | R | G | P | D | A | F | R | F |
| --- | --- | --- | --- | --- | --- | --- | --- | --- | --- | --- | --- | --- | --- | --- | --- | --- | --- | --- | --- | --- | --- | --- | --- | --- | --- | --- | --- | --- | --- | --- | --- | --- | --- | --- | --- | --- | --- | --- | --- | --- |
| Chimpanzee (Pan troglodytes) | M | C | G | I | W | A | L | F | G | S | D | - | - | - | - | - | - | D | C | L | S | V | Q | C | L | S | A | M | K | I | A | H | R | G | P | D | A | F | R | F |
| Sumatran orangutan (Pongo abelii) | M | C | G | I | W | A | L | F | G | S | D | - | - | - | - | - | - | D | C | L | S | V | Q | C | L | S | A | M | K | I | A | H | R | G | P | D | A | F | R | F |
| Cynomolgus monkey (Macaca fascicularis) | M | C | G | I | W | A | L | F | G | S | D | - | - | - | - | - | - | D | C | L | S | V | Q | C | L | S | A | M | K | I | A | H | R | G | P | D | A | F | R | F |
| Rhesus macaque (Macaca mulatta) | M | C | G | I | W | A | L | F | G | S | D | - | - | - | - | - | - | D | C | L | S | V | Q | C | L | S | A | M | K | I | A | H | R | G | P | D | A | F | R | F |
| Mouse (Mus musculus) | M | C | G | I | W | A | L | F | G | S | D | - | - | - | - | - | - | D | C | L | S | V | Q | C | L | S | A | M | K | I | A | H | R | G | P | D | A | F | R | F |
| Rat (Rattus norvegicus) | M | C | G | I | W | A | L | F | G | S | D | - | - | - | - | - | - | D | C | L | S | V | Q | C | L | S | A | M | K | I | A | H | R | G | P | D | A | F | R | F |
| Bovine (Bos taurus) | M | C | G | I | W | A | L | F | G | S | D | - | - | - | - | - | - | D | C | L | S | V | Q | C | L | S | A | M | K | I | A | H | R | G | P | D | A | F | R | F |
| Chicken (Gallus gallus) | M | C | G | I | W | A | L | F | G | S | D | - | - | - | - | - | - | E | C | L | S | V | Q | C | L | S | A | M | K | I | A | H | R | G | P | D | A | F | R | F |
| Western clawed frog (Xenopus tropicalis) | M | C | G | I | W | A | L | F | G | S | D | - | - | - | - | - | - | E | C | L | S | V | Q | C | L | S | A | M | K | I | A | H | R | G | P | D | A | F | R | F |
| Zebrafish (Danio rerio) | M | C | G | I | W | A | L | F | G | S | D | - | - | - | - | - | - | E | C | L | S | V | Q | C | T | N | A | M | K | I | F | H | R | G | P | D | A | F | R | F |
| Fruit fly (Drosophila melanogaster) | M | C | G | I | F | A | I | F | S | R | D | G | E | P | I | P | T | Q | I | L | H | G | S | K | H | S | L | R | E | L | A | Y | R | Q | S | G | K | H | R | H |
|  |  |  |  |  |  |  |  |  |  |  |  |  |  |  |  |  |  |  |  |  |  |  |  |  |  |  |  |  |  |  |  |  |  |  |  |  |  |  |  |  |
| Human (Homo sapiens) | E | N | - | - | - | - | - | - | - | V | N | G | Y | T | N | C | C | F | G | F | H | R | L | A | V | V | D | P | L | F | G | M | Q | P | I | R | V | K | K | Y |
| Chimpanzee (Pan troglodytes) | E | N | - | - | - | - | - | - | - | V | N | G | Y | T | N | C | C | F | G | F | H | R | L | A | V | V | D | P | L | F | G | M | Q | P | I | R | V | K | K | Y |
| Sumatran orangutan (Pongo abelii) | E | N | - | - | - | - | - | - | - | V | N | G | Y | T | N | C | C | F | G | F | H | R | L | A | V | V | D | P | L | F | G | M | Q | P | I | R | V | K | K | Y |
| Cynomolgus monkey (Macaca fascicularis) | E | N | - | - | - | - | - | - | - | V | N | G | Y | T | N | C | C | F | G | F | H | R | L | A | V | V | D | P | L | F | G | M | Q | P | I | R | V | K | K | Y |
| Rhesus macaque (Macaca mulatta) | E | N | - | - | - | - | - | - | - | V | N | G | Y | T | N | C | C | F | G | F | H | R | L | A | V | V | D | P | L | F | G | M | Q | P | I | R | V | K | K | Y |
| Mouse (Mus musculus) | E | N | - | - | - | - | - | - | - | V | N | G | Y | T | N | C | C | F | G | F | H | R | L | A | V | V | D | P | L | F | G | M | Q | P | I | R | V | R | K | Y |
| Rat (Rattus norvegicus) | E | N | - | - | - | - | - | - | - | V | N | G | Y | T | N | C | C | F | G | F | H | R | L | A | V | V | D | P | L | F | G | M | Q | P | I | R | V | R | K | Y |
| Bovine (Bos taurus) | E | N | - | - | - | - | - | - | - | V | N | G | Y | T | N | C | C | F | G | F | H | R | L | A | V | V | D | Q | L | F | G | M | Q | P | I | R | V | K | K | Y |
| Chicken (Gallus gallus) | E | N | - | - | - | - | - | - | - | V | N | G | F | T | N | C | C | F | G | F | H | R | L | A | V | V | D | Q | L | Y | G | M | Q | P | I | R | V | K | K | F |
| Western clawed frog (Xenopus tropicalis) | E | N | - | - | - | - | - | - | - | V | N | G | F | T | N | C | C | F | G | F | H | R | L | A | I | V | D | Q | L | Y | G | M | Q | P | L | R | V | K | K | F |
| Zebrafish (Danio rerio) | E | N | - | - | - | - | - | - | - | V | N | G | F | T | N | C | C | F | G | F | H | R | L | A | I | V | D | Q | L | Y | G | M | Q | P | L | R | V | K | K | F |
| Fruit fly (Drosophila melanogaster) | R | G | P | D | S | T | G | V | Y | V | N | S | L | E | G | V | A | M | I | H | E | R | L | R | I | I | G | V | E | M | G | D | Q | P | F | V | S | E | D | G |
|  |  |  |  |  |  |  |  |  |  |  |  |  |  |  |  |  |  |  |  |  |  |  |  |  |  |  |  |  |  |  |  |  |  |  |  |  |  |  |  |  |
| Human (Homo sapiens) | P | Y | L | W | L | C | Y | N | G | E | I | Y | N | H | K | K | M | Q | Q | H | F | E | F | - | - | - | E | Y | Q | T | K | V | D | G | E | I | I | L | H | L |
| Chimpanzee (Pan troglodytes) | P | Y | L | W | L | C | Y | D | G | E | I | Y | N | H | K | K | M | Q | Q | H | F | E | F | - | - | - | E | Y | Q | T | K | V | D | G | E | I | I | L | H | L |
| Sumatran orangutan (Pongo abelii) | P | Y | L | W | L | C | Y | N | G | E | I | Y | N | H | K | K | M | Q | Q | H | F | E | F | - | - | - | E | Y | Q | T | K | V | D | G | E | I | I | L | H | L |
| Cynomolgus monkey (Macaca fascicularis) | P | Y | L | W | L | C | Y | N | G | E | I | Y | N | Y | K | K | M | Q | R | H | F | E | F | - | - | - | E | Y | Q | T | N | V | D | G | E | I | I | L | H | L |
| Rhesus macaque (Macaca mulatta) | P | Y | L | W | L | C | Y | N | G | E | I | Y | N | Y | K | K | M | Q | R | H | F | E | F | - | - | - | E | Y | Q | T | N | V | D | G | E | I | I | L | H | L |
| Mouse (Mus musculus) | P | Y | L | W | L | C | Y | N | G | E | I | Y | N | H | K | A | L | Q | Q | R | F | E | F | - | - | - | E | Y | Q | T | N | V | D | G | E | I | I | L | H | L |
| Rat (Rattus norvegicus) | P | Y | L | W | L | C | Y | N | G | E | I | Y | N | H | K | A | L | Q | Q | R | F | E | F | - | - | - | E | Y | Q | T | N | V | D | G | E | I | I | L | H | L |
| Bovine (Bos taurus) | P | Y | L | W | L | C | Y | N | G | E | I | Y | N | H | K | K | L | Q | H | H | F | E | F | - | - | - | E | Y | Q | T | K | V | D | G | E | I | I | L | H | L |
| Chicken (Gallus gallus) | P | Y | L | W | L | C | Y | N | G | E | I | Y | N | F | K | Q | L | Q | E | Q | F | G | F | - | - | - | E | Y | Q | T | L | V | D | G | E | V | I | L | H | L |
| Western clawed frog (Xenopus tropicalis) | P | Y | L | W | L | C | Y | N | G | E | I | Y | N | F | K | Q | L | E | K | N | F | G | F | - | - | - | E | Y | Q | T | L | V | D | G | E | V | I | L | H | L |
| Zebrafish (Danio rerio) | P | Y | L | W | L | C | Y | N | G | E | I | Y | N | H | I | K | L | K | N | H | F | E | F | - | - | - | D | Y | Q | T | K | V | D | G | E | I | L | L | H | L |
| Fruit fly (Drosophila melanogaster) | - | N | L | I | L | V | A | N | G | E | I | Y | N | Y | L | E | L | S | A | E | I | A | K | K | R | G | S | Y | N | P | M | S | D | C | H | V | I | L | E | L |
|  |  |  |  |  |  |  |  |  |  |  |  |  |  |  |  |  |  |  |  |  |  |  |  |  |  |  |  |  |  |  |  |  |  |  |  |  |  |  |  |  |
| Human (Homo sapiens) | Y | D | K | G | G | I | E | Q | T | I | C | M | L | D | G | V | F | A | F | V | L | L | D | T | A | N | K | K | V | F | L | G | R | D | T | Y | G | V | R | P |
| Chimpanzee (Pan troglodytes) | Y | D | K | G | G | I | E | Q | T | I | C | M | L | D | G | V | F | A | F | V | L | L | D | T | A | N | K | K | V | F | L | G | R | D | T | Y | G | V | R | P |
| Sumatran orangutan (Pongo abelii) | Y | D | K | G | G | I | E | Q | T | I | C | M | L | D | G | V | F | A | F | V | L | L | D | T | A | T | K | K | V | F | L | G | R | D | T | Y | G | V | R | P |
| Cynomolgus monkey (Macaca fascicularis) | Y | D | K | G | G | I | E | Q | T | I | C | M | L | D | G | V | F | A | F | V | L | L | D | T | A | N | K | K | V | F | L | G | R | D | T | Y | G | V | R | P |
| Rhesus macaque (Macaca mulatta) | Y | D | K | G | G | I | E | Q | T | V | C | M | L | D | G | V | F | A | F | V | L | L | D | T | A | N | K | K | V | F | L | G | R | D | T | Y | G | V | R | P |
| Mouse (Mus musculus) | Y | D | K | G | G | I | E | K | T | I | C | M | L | D | G | V | F | A | F | I | L | L | D | T | A | N | K | K | V | F | L | G | R | D | T | Y | G | V | R | P |
| Rat (Rattus norvegicus) | Y | D | K | G | G | I | E | K | T | I | C | M | L | D | G | V | F | A | F | I | L | L | D | T | A | N | K | K | V | F | L | G | R | D | T | Y | G | V | R | P |
| Bovine (Bos taurus) | Y | D | K | G | G | I | E | Q | T | V | C | M | L | D | G | V | F | A | F | I | L | L | D | T | A | N | K | K | V | F | L | G | R | D | T | Y | G | V | R | P |
| Chicken (Gallus gallus) | Y | N | R | G | G | I | E | Q | T | A | S | M | L | D | G | V | F | A | F | I | L | L | D | T | A | N | R | K | V | F | L | A | R | D | T | Y | G | V | R | P |
| Western clawed frog (Xenopus tropicalis) | Y | S | E | H | G | I | E | K | T | A | A | L | L | D | G | V | F | A | F | I | L | L | D | T | A | N | R | K | V | Y | L | G | R | D | S | Y | G | V | R | P |
| Zebrafish (Danio rerio) | Y | D | R | F | G | I | E | K | M | C | S | L | L | D | G | V | F | A | F | I | L | L | D | T | A | N | R | K | V | H | L | G | R | D | T | Y | G | V | R | P |
| Fruit fly (Drosophila melanogaster) | Y | Q | D | Y | G | - | K | D | L | L | Q | Y | I | T | G | M | F | A | F | A | L | Y | D | R | K | T | K | E | V | L | L | A | R | D | P | F | G | I | I | P |
|  |  |  |  |  |  |  |  |  |  |  |  |  |  |  |  |  |  |  |  |  |  |  |  |  |  |  |  |  |  |  |  |  |  |  |  |  |  |  |  |  |
| Human (Homo sapiens) | L | F | K | A | M | T | E | D | G | F | L | A | V | C | S | E | A | K | G | L | V | T | L | K | H | S | A | T | P | F | L | K | V | E | P | F | L | P | G | H |
| Chimpanzee (Pan troglodytes) | L | F | K | A | M | T | E | D | G | F | L | A | V | C | S | E | A | K | G | L | V | T | L | K | H | S | A | T | P | F | L | K | V | E | P | F | L | P | G | H |
| Sumatran orangutan (Pongo abelii) | L | F | K | A | M | T | E | D | G | F | L | A | V | C | S | E | A | K | G | L | V | T | L | K | H | S | T | T | P | F | L | K | V | E | P | F | L | P | G | H |
| Cynomolgus monkey (Macaca fascicularis) | L | F | K | A | M | T | E | D | G | F | L | A | V | C | S | E | A | K | G | L | V | T | L | K | H | S | T | T | P | F | L | K | V | E | P | F | L | P | G | H |
| Rhesus macaque (Macaca mulatta) | L | F | K | A | M | T | E | D | G | F | L | A | V | C | S | E | A | K | G | L | V | T | L | K | H | S | T | T | P | F | L | K | V | E | P | F | L | P | G | H |
| Mouse (Mus musculus) | L | F | K | A | M | T | E | D | G | F | L | A | V | C | S | E | A | K | G | L | V | S | L | K | H | S | T | T | P | F | L | K | V | E | P | F | L | P | G | H |
| Rat (Rattus norvegicus) | L | F | K | A | L | T | E | D | G | F | L | A | V | C | S | E | A | K | G | L | V | S | L | K | H | S | T | T | P | F | L | K | V | E | P | F | L | P | G | H |
| Bovine (Bos taurus) | L | F | K | A | M | T | E | D | G | F | L | A | V | C | S | E | A | K | G | L | V | N | L | K | H | S | M | T | P | F | L | K | V | E | P | F | L | P | G | H |
| Chicken (Gallus gallus) | L | F | K | V | L | T | D | D | G | F | L | G | V | C | S | E | A | K | G | L | I | N | L | K | H | S | T | S | L | F | P | K | V | E | P | F | L | P | G | H |
| Western clawed frog (Xenopus tropicalis) | L | F | R | L | L | T | D | D | G | F | L | A | V | C | S | E | A | K | G | L | I | D | L | K | H | S | M | T | S | C | P | K | V | D | P | F | P | P | G | H |
| Zebrafish (Danio rerio) | L | F | R | M | L | T | D | D | G | F | L | A | V | C | S | E | G | K | G | L | T | Q | I | K | H | S | M | P | - | T | A | K | I | T | A | F | P | P | G | H |
| Fruit fly (Drosophila melanogaster) | M | Y | V | G | E | D | A | S | G | N | L | W | V | A | S | E | M | K | C | L | V | D | T | - | - | - | - | - | - | C | S | K | V | E | T | F | T | P | G | - |
|  |  |  |  |  |  |  |  |  |  |  |  |  |  |  |  |  |  |  |  |  |  |  |  |  |  |  |  |  |  |  |  |  |  |  |  |  |  |  |  |  |
| Human (Homo sapiens) | Y | E | V | L | D | L | K | P | N | G | K | V | A | S | V | E | M | V | K | Y | H | H | C | R | D | V | P | L | H | A | L | Y | D | N | V | E | K | L | F | P |
| Chimpanzee (Pan troglodytes) | Y | E | V | L | D | L | K | P | N | G | K | V | A | S | V | E | M | V | K | Y | H | H | C | R | D | E | P | L | H | A | L | Y | D | N | V | E | K | L | F | P |
| Sumatran orangutan (Pongo abelii) | Y | E | V | L | D | L | K | P | N | G | K | V | A | S | V | E | M | V | K | Y | H | H | C | R | D | E | P | L | H | A | L | Y | D | N | V | E | K | L | F | P |
| Cynomolgus monkey (Macaca fascicularis) | Y | E | V | L | D | L | K | P | N | G | K | V | A | S | V | E | M | V | K | Y | H | H | C | R | D | E | P | L | H | A | L | Y | D | N | V | D | K | L | F | P |
| Rhesus macaque (Macaca mulatta) | Y | E | V | L | D | L | K | P | N | G | K | V | A | S | V | E | M | V | K | Y | H | H | C | R | D | E | P | L | H | A | L | Y | D | N | V | D | K | L | F | P |
| Mouse (Mus musculus) | Y | E | V | L | D | L | K | P | N | G | K | V | A | S | V | E | M | V | K | Y | H | H | C | T | D | E | P | L | H | A | I | Y | D | S | V | E | K | L | F | P |
| Rat (Rattus norvegicus) | Y | E | V | L | D | L | K | P | N | G | K | V | A | S | V | E | M | V | K | Y | H | H | C | T | D | E | P | L | H | A | I | Y | D | S | V | E | K | L | F | P |
| Bovine (Bos taurus) | Y | E | V | L | D | L | K | P | N | G | K | V | A | S | V | E | M | V | K | H | H | H | C | R | D | E | P | L | H | A | L | Y | D | G | V | E | K | L | F | P |
| Chicken (Gallus gallus) | Y | E | V | L | D | L | K | P | S | G | K | V | V | S | V | E | V | V | K | F | H | S | Y | K | D | E | P | L | H | A | A | C | D | T | V | G | N | L | P | S |
| Western clawed frog (Xenopus tropicalis) | Y | E | V | F | D | L | K | P | S | G | K | V | T | S | V | E | V | I | K | F | H | N | F | R | D | E | P | L | H | A | A | Y | D | T | L | D | K | L | Q | P |
| Zebrafish (Danio rerio) | F | E | V | F | D | L | K | L | N | G | K | V | E | S | V | Q | M | D | R | F | H | C | C | T | D | K | P | K | H | A | D | F | N | K | L | E | G | L | G | T |
| Fruit fly (Drosophila melanogaster) | - | - | E | A | R | F | G | K | V | G | D | F | K | T | C | W | Q | F | Q | Q | S | W | I | K | E | V | P | - | - | - | - | - | - | - | - | - | - | - | - | - |
|  |  |  |  |  |  |  |  |  |  |  |  |  |  |  |  |  |  |  |  |  |  |  |  |  |  |  |  |  |  |  |  |  |  |  |  |  |  |  |  |  |
| Human (Homo sapiens) | G | F | E | I | E | T | V | K | N | N | L | R | I | L | F | N | N | A | V | K | K | R | L | M | T | D | R | R | I | G | C | L | L | S | G | G | L | D | S | S |
| Chimpanzee (Pan troglodytes) | G | F | E | I | E | T | V | K | N | N | L | R | I | L | F | N | N | A | V | K | K | R | L | M | T | D | R | R | I | G | C | L | L | S | G | G | L | D | S | S |
| Sumatran orangutan (Pongo abelii) | G | F | E | I | E | T | V | K | N | N | L | R | I | L | F | N | N | A | V | K | K | R | L | M | T | D | R | R | I | G | C | L | L | S | G | G | L | D | S | S |
| Cynomolgus monkey (Macaca fascicularis) | G | F | E | I | E | T | V | K | N | N | L | R | I | L | F | N | N | A | V | K | K | R | L | M | T | D | R | R | I | G | C | L | L | S | G | G | L | D | S | S |
| Rhesus macaque (Macaca mulatta) | G | F | E | I | E | T | V | K | N | N | L | R | I | L | F | N | N | A | V | K | K | R | L | M | T | D | R | R | I | G | C | L | L | S | G | G | L | D | S | S |
| Mouse (Mus musculus) | G | F | D | L | E | T | V | K | N | N | L | R | I | L | F | D | N | A | I | K | K | R | L | M | T | D | R | R | I | G | C | L | L | S | G | G | L | D | S | S |
| Rat (Rattus norvegicus) | G | F | E | I | E | T | V | K | N | N | L | R | I | L | F | N | N | A | I | K | K | R | L | M | T | D | R | R | I | G | C | L | L | S | G | G | L | D | S | S |
| Bovine (Bos taurus) | G | F | E | I | E | T | V | K | S | N | L | R | I | L | F | D | N | A | V | K | K | R | L | M | T | D | R | R | I | G | C | L | L | S | G | G | L | D | S | S |
| Chicken (Gallus gallus) | G | F | D | L | E | T | V | K | S | N | I | R | V | L | F | E | N | A | V | R | K | R | L | M | A | H | R | R | I | G | C | L | L | S | G | G | L | D | S | S |
| Western clawed frog (Xenopus tropicalis) | G | T | D | R | E | T | V | K | R | N | I | C | C | L | F | E | N | A | V | R | K | R | L | M | A | H | R | R | I | G | C | L | L | S | G | G | L | D | S | S |
| Zebrafish (Danio rerio) | D | F | E | L | E | T | V | K | S | N | I | R | I | L | F | E | D | A | V | R | K | R | L | M | A | H | R | R | I | G | C | L | L | S | G | G | L | D | S | S |
| Fruit fly (Drosophila melanogaster) | - | - | T | Q | T | C | E | L | S | L | L | R | A | N | L | E | F | A | V | R | S | H | L | Q | C | D | V | Q | M | G | A | L | L | S | G | G | V | D | S | S |
|  |  |  |  |  |  |  |  |  |  |  |  |  |  |  |  |  |  |  |  |  |  |  |  |  |  |  |  |  |  |  |  |  |  |  |  |  |  |  |  |  |
| Human (Homo sapiens) | L | V | A | A | T | L | L | K | Q | L | K | E | A | Q | V | Q | Y | P | L | Q | T | F | A | I | G | M | E | D | S | P | D | L | L | A | A | R | K | V | A | D |
| Chimpanzee (Pan troglodytes) | L | V | A | A | T | L | L | K | Q | L | K | E | A | Q | V | Q | Y | P | L | Q | T | F | A | I | G | M | E | D | S | P | D | L | L | A | A | R | K | V | A | D |
| Sumatran orangutan (Pongo abelii) | L | V | A | A | T | L | L | K | Q | L | K | E | A | R | V | Q | Y | P | L | Q | T | F | A | I | G | M | E | D | S | P | D | L | L | A | A | R | K | V | A | D |
| Cynomolgus monkey (Macaca fascicularis) | L | V | A | A | T | L | L | K | Q | L | K | E | A | Q | V | Q | Y | P | L | Q | T | F | A | I | G | M | E | D | S | P | D | L | L | A | A | R | K | V | A | N |
| Rhesus macaque (Macaca mulatta) | L | V | A | A | T | L | L | K | Q | L | K | E | A | Q | V | Q | Y | P | L | Q | T | F | A | I | G | M | E | D | S | P | D | L | L | A | A | R | K | V | A | N |
| Mouse (Mus musculus) | L | V | A | A | S | L | L | K | Q | L | K | E | A | Q | V | Q | Y | P | L | Q | T | F | A | I | G | M | E | D | S | P | D | L | L | A | A | R | K | V | A | N |
| Rat (Rattus norvegicus) | L | V | A | A | S | L | L | K | Q | L | K | E | A | Q | V | P | Y | A | L | Q | T | F | A | I | G | M | E | D | S | P | D | L | L | A | A | R | K | V | A | N |
| Bovine (Bos taurus) | L | V | A | A | T | L | L | K | Q | L | K | E | A | Q | V | Q | Y | P | L | Q | T | F | A | I | G | M | E | D | S | P | D | L | L | A | A | R | K | V | A | N |
| Chicken (Gallus gallus) | L | V | A | A | V | L | L | K | L | M | K | E | M | N | I | K | Y | P | L | Q | T | F | A | I | G | M | E | N | S | P | D | L | L | A | A | R | K | V | A | A |
| Western clawed frog (Xenopus tropicalis) | L | V | A | A | T | L | I | K | L | I | K | E | R | N | M | H | Y | P | L | Q | T | F | A | V | G | T | E | D | S | P | D | L | L | A | A | R | K | V | A | L |
| Zebrafish (Danio rerio) | L | V | A | A | L | L | V | K | L | A | K | E | E | K | L | P | Y | P | I | Q | T | F | S | I | G | A | E | D | S | P | D | V | A | A | A | R | K | V | A | D |
| Fruit fly (Drosophila melanogaster) | L | I | A | S | I | A | T | K | I | M | R | E | R | D | P | N | F | R | L | K | T | F | S | V | G | L | R | D | A | P | D | F | Q | A | A | R | S | V | A | K |
|  |  |  |  |  |  |  |  |  |  |  |  |  |  |  |  |  |  |  |  |  |  |  |  |  |  |  |  |  |  |  |  |  |  |  |  |  |  |  |  |  |
| Human (Homo sapiens) | H | I | G | S | E | H | Y | E | V | L | F | N | S | E | E | G | I | Q | A | L | D | E | V | I | F | S | L | E | T | Y | D | I | T | T | V | R | A | S | V | G |
| Chimpanzee (Pan troglodytes) | H | I | G | S | E | H | Y | E | V | L | F | N | S | E | E | G | I | Q | A | L | D | E | V | I | F | S | L | E | T | Y | D | I | T | T | V | R | A | S | V | G |
| Sumatran orangutan (Pongo abelii) | H | I | G | S | E | H | Y | E | V | L | F | N | S | E | E | G | I | Q | A | L | D | E | V | I | F | S | L | E | T | Y | D | I | T | T | V | R | A | S | V | G |
| Cynomolgus monkey (Macaca fascicularis) | H | I | G | S | E | H | Y | E | V | L | F | N | S | E | E | G | I | Q | A | L | D | E | V | I | F | S | L | E | T | Y | D | I | T | T | V | R | A | S | V | G |
| Rhesus macaque (Macaca mulatta) | H | I | G | S | E | H | Y | E | V | L | F | N | S | E | E | G | I | Q | A | L | D | E | V | I | F | S | L | E | T | Y | D | I | T | T | V | R | A | S | V | G |
| Mouse (Mus musculus) | Y | I | G | S | E | H | H | E | V | L | F | N | S | E | E | G | I | Q | A | L | D | E | V | I | F | S | L | E | T | Y | D | I | T | T | V | R | A | S | V | G |
| Rat (Rattus norvegicus) | Y | I | G | S | E | H | H | E | V | L | F | N | S | E | E | G | I | Q | S | L | D | E | V | I | F | S | L | E | T | Y | D | I | T | T | V | R | A | S | V | G |
| Bovine (Bos taurus) | H | I | G | S | E | H | H | E | V | L | F | N | S | E | E | G | I | Q | V | L | D | E | V | I | F | S | L | E | T | Y | D | I | T | T | V | R | A | S | V | G |
| Chicken (Gallus gallus) | H | I | G | S | E | H | H | E | V | I | F | N | S | E | E | G | I | Q | A | V | E | E | V | I | F | S | L | E | T | Y | D | I | T | T | V | R | A | S | I | G |
| Western clawed frog (Xenopus tropicalis) | H | I | G | S | E | H | H | E | I | M | F | D | P | E | E | G | I | Q | A | V | D | E | V | I | F | S | L | E | T | Y | D | I | T | T | V | R | A | S | V | G |
| Zebrafish (Danio rerio) | Y | I | G | S | E | H | H | V | V | N | F | T | P | E | E | G | I | S | A | L | E | D | V | I | V | H | L | E | S | Y | D | I | T | T | V | R | A | S | I | G |
| Fruit fly (Drosophila melanogaster) | Y | I | D | S | D | H | K | E | I | I | F | E | I | D | E | A | L | D | G | I | R | D | I | I | Y | H | L | E | T | Y | D | V | T | T | V | R | C | S | L | P |
|  |  |  |  |  |  |  |  |  |  |  |  |  |  |  |  |  |  |  |  |  |  |  |  |  |  |  |  |  |  |  |  |  |  |  |  |  |  |  |  |  |
| Human (Homo sapiens) | M | Y | L | I | S | K | Y | I | R | K | N | T | D | S | V | V | I | F | S | G | E | G | S | D | E | L | T | Q | G | Y | I | Y | F | H | K | A | P | S | P | E |
| Chimpanzee (Pan troglodytes) | M | Y | L | I | S | K | Y | I | R | K | N | T | D | S | V | V | I | F | S | G | E | G | S | D | E | L | T | Q | G | Y | I | Y | F | H | K | A | P | S | P | E |
| Sumatran orangutan (Pongo abelii) | M | Y | L | I | S | K | Y | I | R | K | N | T | D | S | V | V | I | F | S | G | E | G | S | D | E | L | T | Q | G | Y | I | Y | F | H | K | A | P | S | P | E |
| Cynomolgus monkey (Macaca fascicularis) | M | Y | L | I | S | K | Y | I | R | K | N | T | D | N | V | V | I | F | S | G | E | G | S | D | E | L | T | Q | G | Y | I | Y | F | H | K | A | P | S | P | E |
| Rhesus macaque (Macaca mulatta) | M | Y | L | I | S | K | Y | I | R | K | N | T | D | N | V | V | I | F | S | G | E | G | S | D | E | L | T | Q | G | Y | I | Y | F | H | K | A | P | S | P | E |
| Mouse (Mus musculus) | M | Y | L | I | S | K | Y | I | R | K | N | T | D | S | V | V | I | F | S | G | E | G | S | D | E | L | T | Q | G | Y | I | Y | F | H | K | A | P | S | P | E |
| Rat (Rattus norvegicus) | M | Y | L | I | S | K | Y | I | R | K | N | T | D | S | V | V | I | F | S | G | E | G | S | D | E | L | T | Q | G | Y | I | Y | F | H | K | A | P | S | P | E |
| Bovine (Bos taurus) | M | Y | L | I | S | K | Y | I | R | K | N | T | D | S | V | V | I | F | S | G | E | G | S | D | E | L | T | Q | G | Y | I | Y | F | H | K | A | P | S | P | E |
| Chicken (Gallus gallus) | M | Y | L | V | S | K | Y | I | R | K | K | T | D | S | V | V | I | F | S | G | E | G | S | D | E | L | T | Q | G | Y | I | Y | F | H | K | A | P | S | P | E |
| Western clawed frog (Xenopus tropicalis) | M | Y | L | I | S | K | Y | I | R | K | K | T | D | S | V | V | I | F | S | G | E | G | S | D | E | L | T | Q | G | Y | I | Y | F | H | K | A | P | S | A | E |
| Zebrafish (Danio rerio) | M | Y | L | V | S | K | Y | I | R | E | K | T | D | S | V | V | I | F | S | G | E | G | S | D | E | L | T | Q | G | Y | I | Y | F | H | K | A | P | S | P | K |
| Fruit fly (Drosophila melanogaster) | M | L | L | L | A | R | Y | I | K | S | - | T | G | I | K | M | I | L | S | G | E | G | A | D | E | I | F | G | G | Y | L | Y | F | H | K | A | P | S | Y | N |
|  |  |  |  |  |  |  |  |  |  |  |  |  |  |  |  |  |  |  |  |  |  |  |  |  |  |  |  |  |  |  |  |  |  |  |  |  |  |  |  |  |
| Human (Homo sapiens) | K | A | E | E | E | S | E | R | L | L | R | E | L | Y | L | F | D | V | L | R | A | D | R | T | T | A | A | H | G | L | E | L | R | V | P | F | L | D | H | R |
| Chimpanzee (Pan troglodytes) | K | A | E | E | E | S | E | R | L | L | R | E | L | Y | L | F | D | V | L | R | A | D | R | T | T | A | A | H | G | L | E | L | R | V | P | F | L | D | H | R |
| Sumatran orangutan (Pongo abelii) | K | A | E | E | E | S | E | R | L | L | R | E | L | Y | L | F | D | V | L | R | A | D | R | T | T | A | A | H | G | L | E | L | R | V | P | F | L | D | H | R |
| Cynomolgus monkey (Macaca fascicularis) | E | A | E | E | E | S | E | R | L | L | R | E | L | Y | L | F | D | V | L | R | A | D | R | T | T | A | A | H | G | L | E | L | R | V | P | F | L | D | H | R |
| Rhesus macaque (Macaca mulatta) | E | A | E | E | E | S | E | R | L | L | R | E | L | Y | L | F | D | V | L | R | A | D | R | T | T | A | A | H | G | L | E | L | R | V | P | F | L | D | H | R |
| Mouse (Mus musculus) | K | A | E | E | E | S | E | R | L | L | K | E | L | Y | L | F | D | V | L | R | A | D | R | T | T | A | A | H | G | L | E | L | R | V | P | F | L | D | H | R |
| Rat (Rattus norvegicus) | K | A | E | E | E | S | E | R | L | L | K | E | L | Y | L | F | D | V | L | R | A | D | R | T | T | A | A | H | G | L | E | L | R | V | P | F | L | D | H | R |
| Bovine (Bos taurus) | K | A | E | E | E | S | E | R | L | L | R | E | L | Y | L | F | D | V | L | R | A | D | R | T | T | A | A | H | G | L | E | L | R | V | P | F | L | D | H | R |
| Chicken (Gallus gallus) | E | A | A | E | E | S | E | R | L | L | K | E | L | Y | L | F | D | V | L | R | A | D | R | T | T | A | A | H | G | L | E | L | R | V | P | F | L | D | H | R |
| Western clawed frog (Xenopus tropicalis) | E | A | A | E | D | S | E | R | L | L | R | E | L | Y | L | F | D | V | L | R | A | D | R | T | T | A | A | H | G | L | E | L | R | V | P | F | L | D | H | R |
| Zebrafish (Danio rerio) | A | G | A | E | D | S | V | R | L | L | E | E | L | Y | L | F | D | V | L | R | A | D | R | T | T | A | A | H | G | L | E | L | R | V | P | F | L | D | H | R |
| Fruit fly (Drosophila melanogaster) | D | F | H | E | E | L | V | K | R | V | R | Q | L | H | L | S | D | C | L | R | A | N | K | V | A | M | A | K | G | V | E | L | R | V | P | F | L | D | T | G |
|  |  |  |  |  |  |  |  |  |  |  |  |  |  |  |  |  |  |  |  |  |  |  |  |  |  |  |  |  |  |  |  |  |  |  |  |  |  |  |  |  |
| Human (Homo sapiens) | F | S | S | Y | Y | L | S | L | P | P | E | M | R | I | P | - | - | - | - | - | - | - | - | - | - | K | N | G | I | E | K | H | L | L | R | E | T | F | E | D |
| Chimpanzee (Pan troglodytes) | F | S | S | Y | Y | L | S | L | P | P | E | M | R | I | P | - | - | - | - | - | - | - | - | - | - | K | N | G | I | E | K | H | L | L | R | E | T | F | E | D |
| Sumatran orangutan (Pongo abelii) | F | S | S | Y | Y | L | S | L | P | P | E | M | R | I | P | - | - | - | - | - | - | - | - | - | - | K | N | G | I | E | K | H | L | L | R | E | T | F | E | D |
| Cynomolgus monkey (Macaca fascicularis) | F | S | S | Y | Y | L | S | L | P | P | E | M | R | T | P | - | - | - | - | - | - | - | - | - | - | K | N | G | I | E | K | H | L | L | R | E | T | F | E | E |
| Rhesus macaque (Macaca mulatta) | F | S | S | Y | Y | L | S | L | P | P | E | M | R | T | P | - | - | - | - | - | - | - | - | - | - | K | N | G | I | E | K | H | L | L | R | E | T | F | E | E |
| Mouse (Mus musculus) | F | S | S | Y | Y | L | S | L | P | P | D | M | R | I | P | - | - | - | - | - | - | - | - | - | - | K | N | G | I | E | K | H | L | L | R | E | T | F | E | D |
| Rat (Rattus norvegicus) | F | S | S | Y | Y | L | S | L | P | P | E | M | R | I | P | - | - | - | - | - | - | - | - | - | - | K | D | G | I | E | K | H | L | L | R | E | T | F | E | D |
| Bovine (Bos taurus) | F | S | S | Y | Y | L | S | L | P | P | D | M | R | V | P | - | - | - | - | - | - | - | - | - | - | K | N | G | I | E | K | H | L | L | R | E | T | F | E | D |
| Chicken (Gallus gallus) | F | T | S | Y | Y | L | S | L | P | A | E | L | R | I | P | - | - | - | - | - | - | - | - | - | - | K | N | G | I | E | K | Y | L | L | R | Q | S | F | E | D |
| Western clawed frog (Xenopus tropicalis) | L | T | A | Y | Y | L | S | L | P | P | E | L | R | I | P | - | - | - | - | - | - | - | - | - | - | K | N | G | I | E | K | Y | L | L | R | E | S | F | E | D |
| Zebrafish (Danio rerio) | F | T | A | Y | Y | L | S | L | P | E | E | M | R | V | P | - | - | - | - | - | - | - | - | - | - | K | N | G | V | E | K | H | L | L | R | D | A | F | D | G |
| Fruit fly (Drosophila melanogaster) | F | V | N | H | V | M | Q | I | R | P | E | D | K | I | P | G | P | L | N | K | F | G | E | V | Q | Q | K | R | L | E | K | Y | V | L | R | A | A | F | A | D |
|  |  |  |  |  |  |  |  |  |  |  |  |  |  |  |  |  |  |  |  |  |  |  |  |  |  |  |  |  |  |  |  |  |  |  |  |  |  |  |  |  |
| Human (Homo sapiens) | S | N | L | I | P | K | E | I | L | W | R | P | K | E | A | F | S | D | G | I | T | S | V | K | N | S | W | F | K | I | L | Q | E | Y | V | E | H | Q | V | D |
| Chimpanzee (Pan troglodytes) | S | N | L | I | P | K | E | I | L | W | R | P | K | E | A | F | S | D | G | I | T | S | V | K | N | S | W | F | K | I | L | Q | E | Y | V | E | H | Q | V | D |
| Sumatran orangutan (Pongo abelii) | S | N | L | I | P | K | E | I | L | W | R | P | K | E | A | F | S | D | G | I | T | S | V | K | N | S | W | F | K | I | L | Q | E | Y | V | E | H | Q | V | D |
| Cynomolgus monkey (Macaca fascicularis) | S | N | L | I | P | K | E | I | L | W | R | P | K | E | A | F | S | D | G | I | T | S | V | K | N | S | W | F | K | I | L | Q | E | Y | V | E | H | Q | V | D |
| Rhesus macaque (Macaca mulatta) | S | N | L | I | P | K | E | I | L | W | R | P | K | E | A | F | S | D | G | I | T | S | V | K | N | S | W | F | K | I | L | Q | E | Y | V | E | H | Q | V | D |
| Mouse (Mus musculus) | C | N | L | L | P | K | E | I | L | W | R | P | K | E | A | F | S | D | G | I | T | S | V | K | N | S | W | F | K | I | L | Q | D | Y | V | E | H | Q | V | D |
| Rat (Rattus norvegicus) | S | N | L | L | P | K | E | I | L | W | R | P | K | E | A | F | S | D | G | I | T | S | V | K | N | S | W | F | K | I | L | Q | D | F | V | E | H | Q | V | D |
| Bovine (Bos taurus) | S | N | L | I | P | K | E | I | L | W | R | P | K | E | A | F | S | D | G | I | T | S | V | K | N | S | W | F | R | I | L | Q | D | Y | I | E | H | Q | V | D |
| Chicken (Gallus gallus) | S | N | L | L | P | K | E | I | L | W | R | P | K | E | A | F | S | D | G | I | A | S | V | K | K | S | W | F | S | I | L | Q | D | Y | I | D | Q | Q | V | D |
| Western clawed frog (Xenopus tropicalis) | S | N | L | L | P | K | D | V | L | W | R | P | K | E | A | F | S | D | G | L | T | S | V | K | K | S | W | F | S | M | L | Q | E | H | I | E | Q | Q | V | D |
| Zebrafish (Danio rerio) | M | N | L | I | P | D | E | I | L | W | R | R | K | E | A | F | S | D | G | L | T | S | V | K | K | S | W | Y | T | S | L | Q | E | H | I | E | S | E | L | N |
| Fruit fly (Drosophila melanogaster) | - | N | Y | L | P | D | E | V | L | W | R | Q | K | E | Q | F | S | D | G | V | G | - | - | - | Y | D | W | I | D | S | I | R | R | V | A | T | S | H | V | S |
|  |  |  |  |  |  |  |  |  |  |  |  |  |  |  |  |  |  |  |  |  |  |  |  |  |  |  |  |  |  |  |  |  |  |  |  |  |  |  |  |  |
| Human (Homo sapiens) | D | A | M | M | A | N | A | A | Q | K | F | P | F | N | T | P | K | T | K | E | G | Y | Y | Y | R | Q | V | F | E | R | H | Y | P | G | R | - | A | D | W | L |
| Chimpanzee (Pan troglodytes) | D | A | M | M | A | N | A | A | Q | K | F | P | F | N | T | P | K | T | K | E | G | Y | Y | Y | R | Q | V | F | E | R | H | Y | P | G | R | - | A | D | W | L |
| Sumatran orangutan (Pongo abelii) | D | A | M | M | A | N | A | A | Q | K | F | P | F | N | T | P | K | T | K | E | G | Y | Y | Y | R | Q | V | F | E | R | H | Y | P | G | R | - | A | D | W | L |
| Cynomolgus monkey (Macaca fascicularis) | D | A | M | M | A | N | A | A | Q | K | F | P | F | N | T | P | K | T | K | E | G | Y | Y | Y | R | Q | I | F | E | R | H | Y | P | G | R | - | A | D | W | L |
| Rhesus macaque (Macaca mulatta) | D | A | M | M | A | N | A | A | Q | K | F | P | F | N | T | P | K | T | K | E | G | Y | Y | Y | R | Q | I | F | E | R | H | Y | P | G | R | - | A | D | W | L |
| Mouse (Mus musculus) | D | E | M | M | S | A | A | S | Q | K | F | P | F | N | T | P | K | T | K | E | G | Y | F | Y | R | Q | I | F | E | R | H | Y | P | G | R | - | A | D | W | L |
| Rat (Rattus norvegicus) | D | A | M | M | S | E | A | S | Q | K | F | P | F | N | T | P | Q | T | K | E | G | Y | Y | Y | R | Q | I | F | E | H | H | Y | P | G | R | - | A | D | W | L |
| Bovine (Bos taurus) | D | A | A | M | A | S | A | A | Q | K | F | P | I | N | T | P | K | T | K | E | G | Y | Y | Y | R | Q | I | F | E | N | H | Y | P | G | R | - | A | D | W | L |
| Chicken (Gallus gallus) | D | L | L | L | E | K | A | A | E | K | Y | P | F | N | P | P | R | T | K | E | S | Y | Y | Y | R | Q | I | F | E | K | H | Y | P | G | R | - | S | S | W | L |
| Western clawed frog (Xenopus tropicalis) | N | I | V | L | E | K | A | A | E | N | F | P | F | N | P | P | K | T | K | E | G | Y | F | Y | R | Q | I | F | E | K | Y | Y | P | G | R | - | A | G | W | L |
| Zebrafish (Danio rerio) | D | S | Q | L | E | N | A | N | K | L | Y | H | I | N | P | P | T | T | K | E | G | L | F | I | R | Q | I | F | E | K | H | Y | P | G | Q | - | G | E | W | T |
| Fruit fly (Drosophila melanogaster) | D | Q | E | F | S | A | A | A | L | R | F | P | F | N | T | P | T | T | K | E | A | F | Y | Y | R | C | I | F | A | E | Q | F | P | G | E | S | A | A | R | T |
|  |  |  |  |  |  |  |  |  |  |  |  |  |  |  |  |  |  |  |  |  |  |  |  |  |  |  |  |  |  |  |  |  |  |  |  |  |  |  |  |  |
| Human (Homo sapiens) | S | H | Y | W | M | P | - | - | K | W | I | N | A | T | D | P | S | A | R | T | L | T | H | Y | K | S | A | V | K | A |  |  |  |  |  |  |  |  |  |  |
| Chimpanzee (Pan troglodytes) | S | H | Y | W | M | P | - | - | K | W | I | N | A | T | D | P | S | A | R | T | L | T | H | Y | K | S | A | V | K | A |  |  |  |  |  |  |  |  |  |  |
| Sumatran orangutan (Pongo abelii) | S | H | Y | W | M | P | - | - | K | W | I | N | A | T | D | P | S | A | R | T | L | T | H | Y | K | S | A | V | K | A |  |  |  |  |  |  |  |  |  |  |
| Cynomolgus monkey (Macaca fascicularis) | S | H | Y | W | M | P | - | - | K | W | I | N | A | T | D | P | S | A | R | T | L | T | H | Y | K | S | A | A | K | A |  |  |  |  |  |  |  |  |  |  |
| Rhesus macaque (Macaca mulatta) | S | H | Y | W | M | P | - | - | K | W | I | N | A | T | D | P | S | A | R | T | L | T | H | Y | K | S | A | A | K | A |  |  |  |  |  |  |  |  |  |  |
| Mouse (Mus musculus) | T | H | Y | W | M | P | - | - | K | W | I | N | A | T | D | P | S | A | R | T | L | T | H | Y | K | S | A | A | K | A |  |  |  |  |  |  |  |  |  |  |
| Rat (Rattus norvegicus) | T | H | Y | W | M | P | - | - | K | W | I | N | A | T | D | P | S | A | R | T | L | T | H | Y | K | S | T | A | K | A |  |  |  |  |  |  |  |  |  |  |
| Bovine (Bos taurus) | P | H | Y | W | M | P | - | - | R | W | T | N | A | T | D | P | S | A | R | T | L | T | H | Y | K | A | A | A | K | A |  |  |  |  |  |  |  |  |  |  |
| Chicken (Gallus gallus) | P | H | Y | W | M | P | - | - | R | W | V | E | A | T | D | P | S | A | R | T | L | K | H | Y | K | S | A | I | Q | E |  |  |  |  |  |  |  |  |  |  |
| Western clawed frog (Xenopus tropicalis) | T | H | Y | W | M | P | - | - | R | W | I | S | A | T | D | P | S | A | R | T | L | K | H | Y | K | S | D | T | N | E |  |  |  |  |  |  |  |  |  |  |
| Zebrafish (Danio rerio) | P | H | Y | W | M | P | - | - | R | W | I | K | A | T | D | P | S | A | R | T | L | S | I | Y | K | P | D | K | E | Q |  |  |  |  |  |  |  |  |  |  |
| Fruit fly (Drosophila melanogaster) | V | V | R | W | V | P | R | L | D | W | G | C | P | E | D | P | S | G | R | A | Q | A | V | H | Q | V | N | K | N | - |  |  |  |  |  |  |  |  |  |  |

Supplementary table 1: The complete conservation analysis of ASNS protein sequences across 12 different species. Conservation analysis was performed in MEGA7 using ClustalW algorithm with default settings. The mutation sites within the patient are highlighted in orange which are shown to be highly conserved across all the listed species.
